# Enhanced axonal mitochondrial motility and neural activity-induced energy deficits destabilize synaptic transmission in models of bipolar disorder

**DOI:** 10.1038/s41467-026-76722-x

**Published:** 2026-08-22

**Authors:** Sunan Li, Gui-Jing Xiong, Zezhi Li, Zu-Hang Sheng

**Affiliations:** Synaptic Function Section, The Porter Neuroscience Research Center, National Institute of Neurological Disorders and Stroke (NINDS), National Institutes of Health, Bethesda, MD USA

**Keywords:** Cellular neuroscience, Bipolar disorder

## Abstract

Synaptic communication requires mitochondria to supply ATP and buffer calcium at presynaptic terminals. In bipolar disorder, manic episodes are associated with elevated mood and neural activity, but the underlying cellular mechanisms remain unclear. Here we show that hiPSC-derived cortical neurons from donors with bipolar disorder exhibit increased axonal mitochondrial motility and frequent mitochondrial entry–exit transitions, reducing stable mitochondrial retention at presynaptic terminals. This destabilizes local ATP maintenance and calcium buffering, increasing synaptic variability without altering mean synaptic strength. Knockdown of the bipolar disorder risk gene *AKAP11* in mouse neurons reproduced these synaptoenergetic deficits. HiPSC-derived neurons from donors with bipolar disorder exhibited reduced expression of the mitochondrial anchor protein syntaphilin(SNPH), and  *snph *knockout mice displayed manic-like behavioral phenotypes. Lithium restored presynaptic mitochondrial retention, improved ATP maintenance, rescued synaptic variability, and reversed behavioral phenotypes. These findings support impaired presynaptic mitochondrial retention and activity-induced synaptoenergetic deficits as cellular mechanisms contributing to bipolar disorder.

## Abstract (web summary)

The contribution of mitochondrial trafficking and positioning to the disease mechanisms of bipolar disorder is unclear. Li et al. show that axonal mitochondrial hypermotility destabilizes presynaptic energy homeostasis, increasing synaptic variability in models of bipolar disorder that can be rescued by lithium treatment.

## Introduction

Neurons communicate through synaptic transmission, a process that underlies cognition, mood, and movement. This communication imposes substantial bioenergetic demands, requiring adenosine triphosphate (ATP) generated primarily through glycolysis and mitochondrial oxidative phosphorylation (OxPhos)^1,2^. ATP fuels virtually every step of synaptic transmission, including maintenance of the resting membrane potentials, generation of action potentials, and synaptic vesicle (SV) recycling, encompassing exocytosis, endocytosis, neurotransmitter refilling, and vesicle mobilization^1,3,4^. Together, these energy-dependent processes sustain synaptic efficacy and plasticity. Approximately 55% of neuronal ATP is consumed within axons, where more than 20,000 ATP molecules are required to support a single cycle of glutamatergic SV recycling^1,5,6^. Mitochondria supply up to 93% of the ATP consumed at synapses^3,7^. Because synaptic activity and mitochondrial trafficking are both highly dynamic, local ATP availability must be continuously matched to fluctuating energy demands to maintain efficient synaptic transmission^8–11^. However, only 33–50% of presynaptic terminals in the central nervous system (CNS) contain resident mitochondria^12–14^, limiting local ATP production and rendering synapses particularly susceptible to activity-induced energy deficits. Consequently, mechanisms that dynamically adapt synaptic energy metabolism are essential for maintaining stable synaptic transmission, whereas their disruption may compromise synaptic function and contribute to cognitive and mood disorders^1,9,10,14–19^. Defining how synapses dynamically regulate local energy metabolism—and how failures of this process contribute to synaptic variability—may provide new insights into the pathophysiology of mood disorders.

Bipolar disorder (BD) is a severe psychiatric disorder characterized by recurrent episodes of mania and depression. Mania, the defining feature of bipolar I disorder, is marked by elevated mood, increased arousal, and heightened psychomotor activity, and affects approximately 0.6–1.0% of the global population over a lifetime. Genome-wide association studies have implicated genetic variants involved in glutamatergic neurotransmission, calcium signaling, and hormonal regulation in BD susceptibility^20^. However, progress toward understanding disease mechanisms has been hindered by the limited availability of robust cellular and animal models. Analyses of postmortem brain tissue and patient-derived neurons have identified alterations in mitochondrial bioenergetics and dynamics, including changes in mitochondrial DNA (mtDNA) copy number, mitochondria abundance, Ca^2+^ signaling, and metabolic remodeling toward greater reliance on glycolysis at the expense of oxidative phosphorylation^21–36^. Consistent with these findings, synaptic dysfunction within the prefrontal cortex, hippocampus, and amygdala is a hallmark of BD^37,38^, and neurons derived from patients with BD exhibit increased energetic demands to sustain glutamatergic neurotransmission^15,35,36,39,40^. Despite these observations, mitochondrial dysfunction is typically associated with global energy failure and progressive neurodegeneration—outcomes that contrast with the episodic and reversible mood disturbances. This discrepancy raises the possibility that subtle defects in activity-dependent mitochondrial support, rather than overt bioenergetic failure, contribute to disease pathogenesis. However, whether impaired presynaptic mitochondrial support disrupts synaptic transmission and contributes to mood instability remains unknown. Lithium remains a first-line treatment of bipolar disorder, but its cellular mechanisms of action are incompletely understood^41,42^. Defining how presynaptic energy metabolism is altered in bipolar disorder may therefore provide mechanistic insights into disease pathogenesis and inform therapeutic strategies to restore synaptic energy homeostasis.

Here, we establish two complementary models of bipolar disorder: human iPSC-derived cortical neurons from donors with bipolar disorder and mouse cortical neurons with reduced expression of AKAP11, a genetic risk factor for BD^43^. Despite preserved mitochondrial membrane potential and bioenergetic capacity, both neuronal models exhibit axonal mitochondrial hypermotility, resulting in frequent mitochondrial entry–exit transitions at presynaptic terminals. These dynamic transitions destabilize local ATP availability and Ca^2+^ buffering, increasing pulse-to-pulse variability in synaptic transmission. In hiPSC-derived cortical neurons from donors with bipolar disorder, these phenotypes are associated with reduced expression of syntaphilin (SNPH), an axonal mitochondrial anchor that retains mitochondria at presynaptic terminals^9,10,12^. Consistent with this finding, *Snph* knockout mice display mania-like behaviors that are attenuated by chronic lithium treatment. Mechanistically, lithium restores presynaptic mitochondrial retention by enhancing Ca^2+^ efflux through the Na^+^/Ca^2+^/Li^+^ exchanger (NCLX), thereby sustaining Miro1-dependent Ca^2+^ signaling in arresting motile mitochondria and stabilizing local ATP supply. Restoration of presynaptic mitochondrial retention reduces synaptic variability. Together, our findings identify dysregulated mitochondrial positioning as a mechanism associated with synaptic variability and mood-related behavioral abnormalities and provide a cellular framework for understanding how lithium restores presynaptic mitochondrial support.

## Results

### Activity-induced presynaptic energy deficits in hiPSC-derived neurons from donors with bipolar disorder

**Fig. 1:**
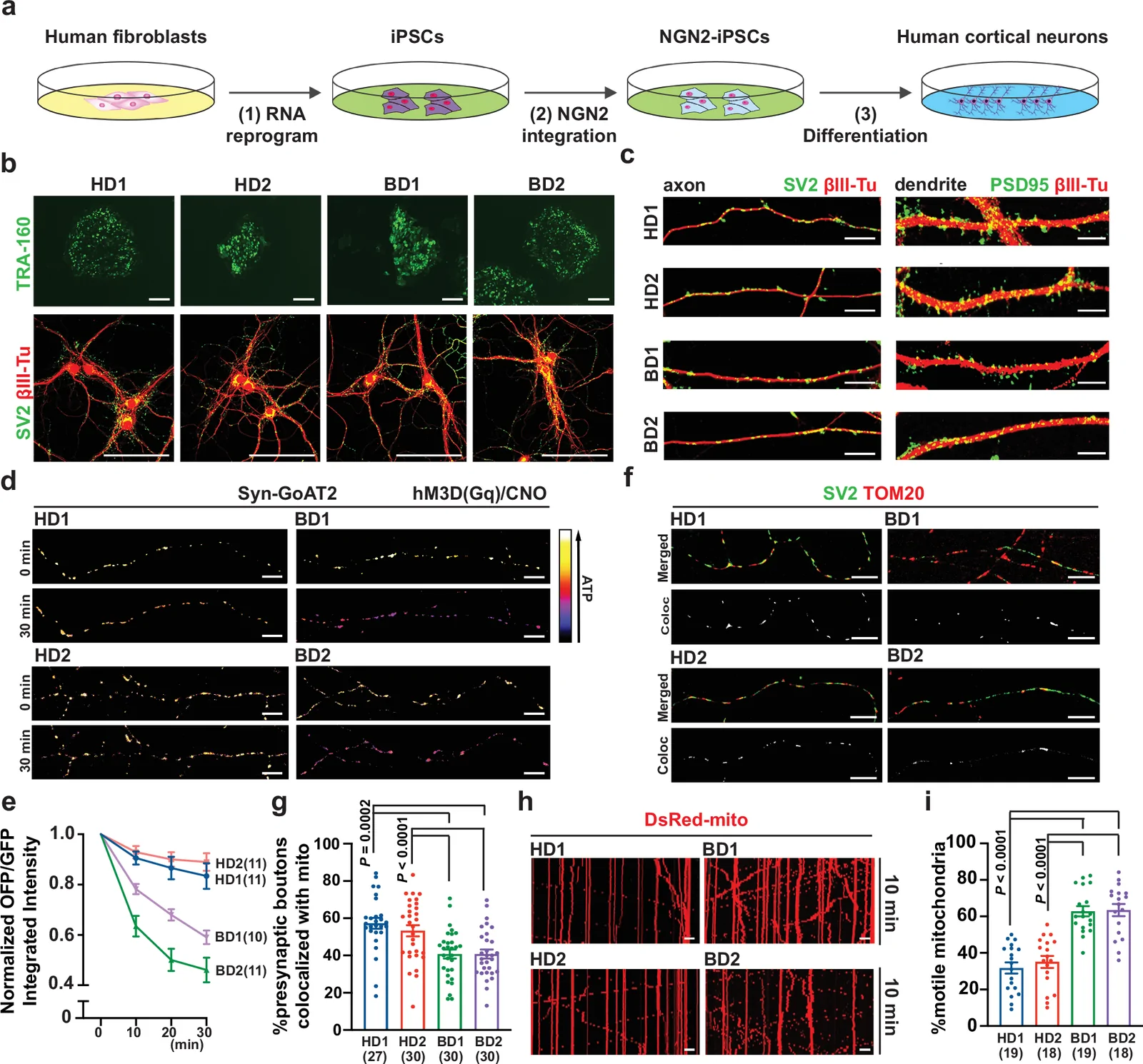
hiPSC-derived neurons from donors with bipolar disorder display activity-induced presynaptic energy deficits. **a** Schematic illustrating generation of hiPSC-derived cortical neurons. Fibroblasts from healthy donors (HD) and donors with bipolar disorder (BD) were reprogrammed into hiPSCs, engineering with a doxycycline-inducible NGN2 cassette, and differentiated into cortical neurons. **b**, **c** Characterization of hiPSC-derived neurons. TRA-1-60 immunostaining confirms pluripotency of the hiPSC lines (**b**, upper). At day 21 post-induction (DPI21), cortical neurons were immunostained for β3-tubulin (β3-Tu) together with SV2 or PSD95. The images document the generation of iPSC-derived cortical neurons and therefore were not repeated. However, the validation of each iPSC line were performed independently more than three times. Representative images (**d**) and quantification (**e**) showing activity-induced presynaptic ATP decline in neurons from donors with bipolar disorder compared with healthy donors. Neurons expressing hM3D(Gq) and the presynaptic ATP sensor Syn-GoATeam2 were imaged at DPI21 before and after stimulation with 10 µM clozapine-N-oxide (CNO). ATP levels are displayed as OFP/GFP ratio heatmaps. The pseudocolor bar scale represents fluorescence intensity in arbitrary units (AU), with blue indicating low ATP and red indicating high ATP. Two-way ANOVA: cell line, *F*_*1, 41*_ = 53.84, *P* < 0.0001; time x cell line, *F*_*3, 123*_ = 39.78, *P* < 0.0001). **f**, **g** Reduced presynaptic mitochondrial retention in hiPSC-derived neurons from donors with bipolar disorder. Neurons were co-immunostained for SV2 and TOM20. Colocalized pixels (white in binary images) indicate presynaptic mitochondria. **h**, **i** Increased axonal mitochondrial motility in neurons from donors with bipolar disorder. Neurons expressing DsRed-Mito were subjected to 10-min time-lapse imaging at DPI21. In kymographs, vertical lines represent stationary mitochondria, whereas diagonal or curved lines indicate motile mitochondria. Neurons from donors with bipolar disorder displayed a 50% increase in axonal mitochondrial motility compared neurons from healthy donors (all comparisons, *P* < 0.0001). Data are presented as mean ± s.e.m. from the total number of neurons indicated in parentheses across three independent experiments and were analyzed by two-way ANOVA (**e**) or one-way ANOVA with Tukey’s post-hoc test (**g**, **i**). Scale bars: 100 µm (**b**); 10 µm (**c**, **d**, **f**, **h**). Source data are provided as a Source Data file.

Approximately 40% of individuals with bipolar disorder exhibit metabolic syndrome^27,33,34,44^, highlighting a potential link between systemic metabolic dysfunction and the high energy demands of synaptic transmission, a process critical for mood regulation^3,45^. We hypothesized that impaired presynaptic mitochondrial support contributes to altered synaptic function associated with bipolar disorder. Because no single genetic model fully recapitulates bipolar disorder-related behavioral phenotypes^46^, we generated hiPSC-derived cortical neurons from primary fibroblasts obtained from donors with bipolar disorder and healthy family-matched control donors (Coriell Institute) (Supplementary Table 1). Fibroblasts were reprogrammed using a non-integrating mRNA-based approach to generate three bipolar disorder lines (BD1–BD3) and two healthy donor lines (HD1 and HD2) (Fig. 1a). The resulting hiPSCs expressed the pluripotency marker TRA-1-60 (Fig. 1b).

Cortical neuronal differentiation was induced by integrating a doxycycline-inducible *neurogenin 2* (NGN2) transgene using the PiggyBac system, followed by doxycycline treatment for 4 days^47,48^. By 21 days post-induction (DPI21), neurons expressed the neuronal marker β3-tubulin and the synaptic markers SV2 and PSD95, consistent with neuronal differentiation and synapse formation (Fig. 1b, c and Supplementary Fig. 1a). Synapse density increased significantly between DPI17 and DPI34, indicating progressive synaptic maturation, with no significant differences between neurons derived from healthy donors and donors with bipolar disorder at either time point (Supplementary Fig. 1b, c). These findings indicate that mature hiPSC-derived cortical neurons provide an appropriate bipolar disorder model for examining presynaptic energy homeostasis and synaptic function.

To determine whether neurons from donors with bipolar disorder exhibit impaired presynaptic energy maintenance during sustained activity, we used a chemogenetic stimulation paradigm. Cortical neurons expressing the Gq-coupled designer receptor hM3D(Gq) showed increased c-Fos expression and enhanced neuronal firing following clozapine-N-oxide (CNO) treatment^49,50^, confirming effective neuronal activation (Supplementary Fig. 2a–d). Presynaptic ATP levels were monitored using the synaptic vesicle-targeted ATP sensor Syn-GoATeam2 and quantified as the OFP/GFP fluorescence ratio (Fig. 1d). Following a 10-min CNO pre-stimulation, hiPSC-derived cortical neurons from donors with bipolar disorder exhibited a more rapid decline in presynaptic ATP during sustained stimulation than neurons from healthy donors. Significant differences were detected at both 20 min (HD1 vs. BD1, *P* = 0.0002; HD1 vs. BD2, *P* = 0.0105; HD2 vs. BD1, *P* < 0.0001; HD2 vs. BD2, *P* < 0.0001) and 30 min (HD1 vs. BD1, *P* = 0.0005; HD1 vs. BD2, *P* = 0.0036; HD2 vs. BD1, *P* < 0.0001; HD2 vs. BD2, *P* < 0.0001) of stimulation (Fig. 1e). These findings indicate that hiPSC-derived neurons from donors with bipolar disorder exhibit reduced capacity to maintain presynaptic ATP during sustained neuronal activity, consistent with impaired presynaptic energy homeostasis.

### Reduced presynaptic mitochondrial retention in hiPSC-derived neurons from donors with bipolar disorder

Presynaptic mitochondria provide local ATP to sustain synaptic transmission during prolonged neuronal activity^9,10^. To determine whether the presynaptic energy deficits observed in hiPSC-derived neurons from donors with bipolar disorder are associated with impaired presynaptic mitochondrial retention, we examined mitochondrial distribution by co-immunostaining for the synaptic vesicle marker SV2 and the mitochondrial marker TOM20. Mitochondria were detected at 57.27% and 60.10% of presynaptic terminals in neurons from healthy donors (HD1 and HD2), compared with 40.78% and 40.81% in neurons from donors with bipolar disorder (BD1 and BD2) (HD1 vs. BD1, *P* = 0.0002; HD1 vs. BD2, *P* = 0.0002; HD2 vs. BD1, *P* ≤ 0.0001; HD2 vs. BD2, *P* ≤ 0.0001) (Fig. 1f, g). Time-lapse imaging further showed an approximately 50% increase in axonal mitochondrial motility in neurons from donors with bipolar disorder compared with neurons from healthy donors (all comparisons, *P* < 0.0001) (Fig. 1h, i).

The reduction in presynaptic mitochondrial occupancy likely underestimates the functional deficit because motile mitochondria can transiently pass through presynaptic sites and be scored as presynaptic mitochondria in fixed-cell analyses. Such transiently localized mitochondria are unlikely to provide sustained ATP production during prolonged synaptic activity. Consistent with this interpretation, neurons from donors with bipolar disorder exhibited both reduced presynaptic mitochondrial occupancy and accelerated presynaptic ATP depletion during sustained stimulation (Fig. 1e), whereas neurons from healthy donors maintained higher presynaptic ATP levels. These findings are consistent with increased axonal mitochondrial motility reducing mitochondrial retention at presynaptic terminals and compromising presynaptic energy maintenance.

Axonal mitochondria undergo bidirectional transport, and their motility is tightly regulated during neuronal maturation, primarily through developmentally controlled expression of syntaphilin (SNPH), an axonal mitochondrial anchoring protein. Increased SNPH expression during neuronal maturation is associated with a progressive decline in axonal mitochondrial motility—from ~50% at DIV7 to ~20% by DIV18^10,51^. Because mitochondria are preferentially retained at sites of high energy demand, such as presynaptic terminals^52^, disruption of this anchoring mechanism increases mitochondrial motility and reduces presynaptic mitochondrial retention^10^. Together, these findings support a model in which axonal mitochondrial hypermotility compromises mitochondrial retention at presynaptic terminals and, therefore, impaired energy homeostasis during sustained synaptic activity in hiPSC-derived neurons from donors with bipolar disorder.

### Human iPSC-derived cortical neurons from donors with bipolar disorder exhibit increased synaptic variability

**Fig. 2:**
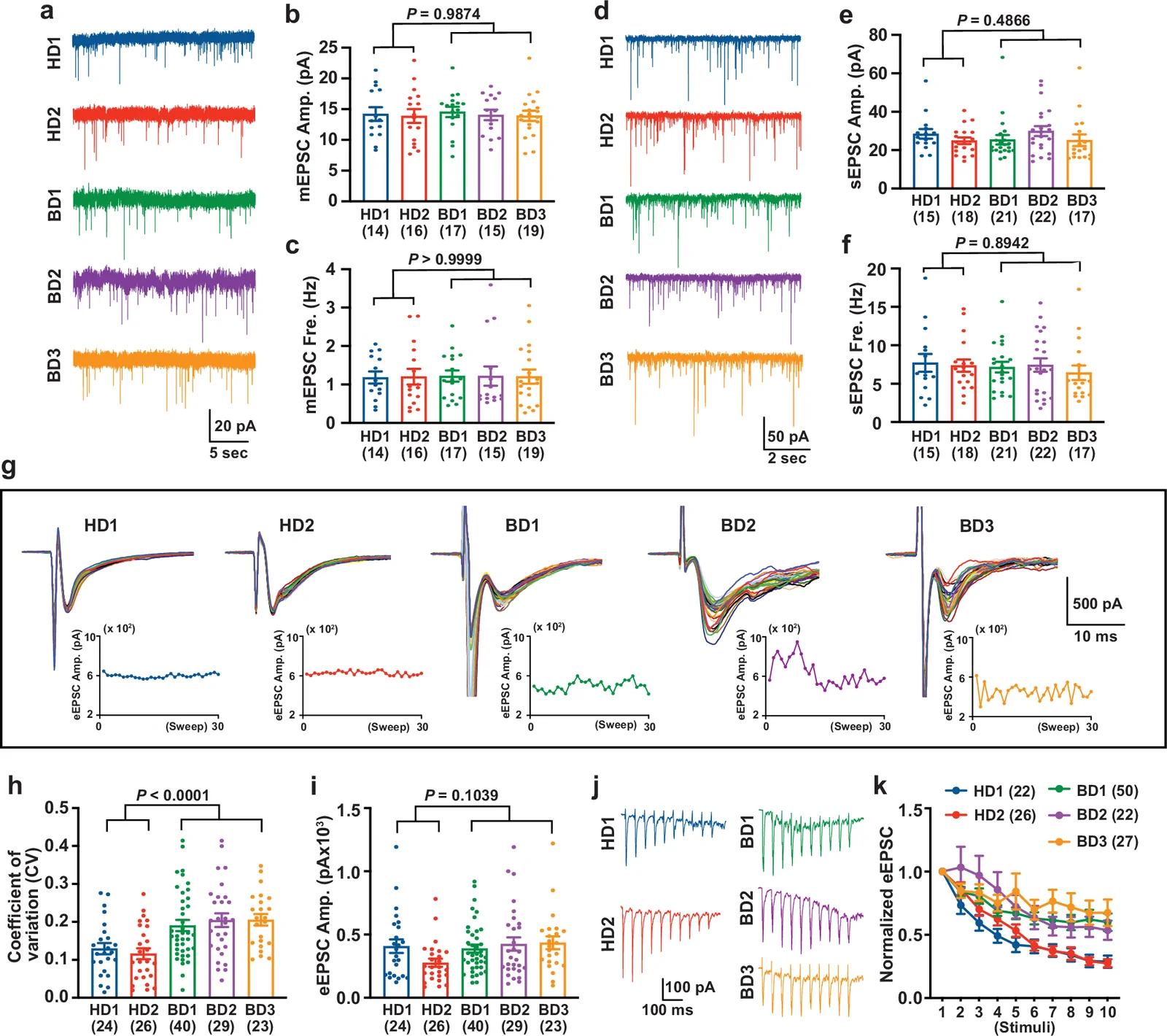
hiPSC-derived neurons from donors with bipolar disorder exhibit increased synaptic variability. Representative miniature excitatory postsynaptic current (mEPSC) traces (**a**) and quantification of mEPSC amplitude (**b**) and frequency (**c**) recorded from hiPSC-derived cortical neurons at DPI34-38 using whole-cell patch-clamp recordings in the presence of 1 μM tetrodotoxin (TTX). No significant differences were detected in mean mEPSC amplitude or frequency among neurons derived from two healthy donors (HD) and three donors with bipolar disorder (one-way ANOVA: amplitude, *F*_*4,76*_ = 0.083, *P* = 0.9874; frequency, *F*_*4,76*_ = 0.0059, *P* > 0.9999). Representative spontaneous EPSC (sEPSC) traces (**d**) and quantification of sEPSC amplitude (**e**) and frequency (**f**) recorded in the absence of TTX. No significant differences were observed among the five cell lines (one-way ANOVA: amplitude, *F*_*4,88*_ = 0.8679, *P* = 0.4866; frequency, *F*_*4,88*_ = 0.2738, *P* = 0.8942). **g**–**i** Increased pulse-to-pulse variability in evoked EPSC (eEPSC) amplitudes in neurons from donors with bipolar disorder. **g** Superimposed traces of 30 consecutive eEPSC sweeps recorded at 0.05 Hz. **h** Coefficient of variation (CV) of eEPSC amplitudes. **i** mean eEPSC peak amplitudes. Neurons from donors with bipolar disorder exhibited a significantly higher CV than healthy donor neurons (one-way ANOVA: *F*_*4,137*_ = 6.821, *P* < 0.0001), whereas mean eEPSC amplitude was unchanged (one-way ANOVA: *F*_*4,137*_ = 1.961, *P* = 0.1039). **j**, **k** Altered short-term synaptic depression in neurons from donors with bipolar disorder. **j** Representative eEPSC traces evoked at 20-Hz stimulation for 500 ms. **k** Normalized eEPSC amplitudes plotted against the first response. Two-way ANOVA revealed a significant effect of cell line (*F*_*4,142*_ = 4.461, *P* = 0.0020) and a significant interaction between stimulus number and cell lines (*F*_*6.222,220.9*_ = 2.971, *P* = 0.0075). Data are presented as mean ± s.e.m. from the number of neurons indicated in parentheses across at least three independent experiments and were analyzed using one-way ANOVA with Tukey’s post-hoc test (**b**, **c**, **e**, **f**, **h**, **i**), or two-way ANOVA with Holm–Šidák multiple-comparison tests (**k**). Source data are provided as a Source Data file.

Because synaptic transmission is a highly energy-demanding process^1,3,9,14^, we next examined whether activity-induced energy deficits alter synaptic transmission in hiPSC-derived cortical neurons. Whole-cell patch-clamp recordings were performed at DPI30-38 (Supplementary Fig. 2e). To assess functional synaptic connectivity, we recorded miniature (mEPSCs) and spontaneous (sEPSCs) excitatory postsynaptic currents. In the presence of tetrodotoxin (TTX), mean mEPSC amplitude and frequency did not differ among neurons from healthy donors and donors with bipolar disorder (one-way ANOVA, amplitude: *F*_*4,76*_ = 0.083, *P* = 0.9874; frequency: *F*_*4,76*_ = 0.0059, *P* > 0.9999) (Fig. 2a–c), suggesting comparable postsynaptic AMPA receptor function and presynaptic release sites. Likewise, sEPSC amplitude and frequency were also comparable across groups (one-way ANOVA, amplitude: *F*_*4,88*_ = 0.8679, *P* = 0.4866; frequency: *F*_*4,88*_ = 0.2738, *P* = 0.8942) (Fig. 2d–f), indicating similar baseline synaptic connectivity and excitability.

We next assessed paired-pulse ratio (PPR), an indicator of presynaptic release probability, using a 50-ms interstimulus interval. Although mean PPR was similar between groups, hiPSC-derived neurons from donors with bipolar disorder exhibited significantly greater sweep-to-sweep variability, reflected by an increased coefficient of variation (CV; *P* = 0.0002) (Supplementary Fig. 2f–i). This increased variability appeared to arise from fluctuations in the first eEPSC that propagated to the second response. To further examine synaptic stability, evoked EPSCs (eEPSCs) were recorded over 30 consecutive sweeps at 0.05 Hz. Mean eEPSC amplitudes were comparable between groups (Fig. 2g–i). In contrast, all three bipolar disorder lines (BD1-BD3) consistently displayed increased pulse-to-pulse variability in eEPSC amplitude, as indicated by a higher CV (HD vs BD, *P* < 0.0001, Fig. 2h). Thus, despite preserved mean synaptic strength, hiPSC-derived neurons from donors with bipolar disorder exhibited greater temporal variability in synaptic transmission. Given the increased axonal mitochondrial motility observed across iPSC-derived BD lines, these results are consistent with unstable presynaptic ATP supply at presynaptic terminals driving elevated synaptic variability.

Presynaptic mitochondria support synaptic transmission by supplying ATP and buffering intracellular Ca^2+^^53,54^, two processes required for short-term synaptic plasticity^12,55,56^. Impaired mitochondrial retention not only reduces local ATP supply required for driving synaptic vesicle recycling but also leads to elevated presynaptic [Ca^2+^]_i_ transients during repetitive stimulation, contributing to slower short-term synaptic depression^10^. To determine whether altered mitochondrial retention affects short-term depression in hiPSC-derived neurons, we recorded evoked EPSCs during 20-Hz, 500 ms stimulation trains. Under 3 mM extracellular Ca^2+^, neurons from donors with bipolar disorder exhibited slower synaptic depression than neurons from healthy donors (Fig. 2j, k; two-way ANOVA, group effect: *F*_*4,142*_ = 4.461, *P* = 0.0020). We then repeated the experiments under a physiological extracellular Ca^2+^ concentration (1.3 mM)^57^. Individual bipolar disorder lines showed a similar trend but did not reach statistical significance (two-way ANOVA, main effect of cell line, *P* = 0.2281; stimulus × cell line interaction, *P* = 0.0992) (Supplementary Fig. 2j, k). When data were pooled by group, however, neurons from donors with bipolar disorder exhibited significantly slower short-term synaptic depression than neurons from healthy donors (Supplementary Fig. 2l; two-way ANOVA, group effect, *P* = 0.0193; stimulus × group interaction, *P* = 0.0018). These findings are consistent with reduced presynaptic mitochondrial retention contributing to altered presynaptic Ca^2+^ handling and short-term synaptic plasticity in hiPSC-derived neurons from donors with bipolar disorder under both elevated (3 mM) and physiological (1.3 mM) extracellular Ca^2+^ conditions, supporting the physiological relevance of this phenotype.

### Increased mitochondrial transit events at individual presynapses in hiPSC-derived neurons from donors with bipolar disorder

**Fig. 3:**
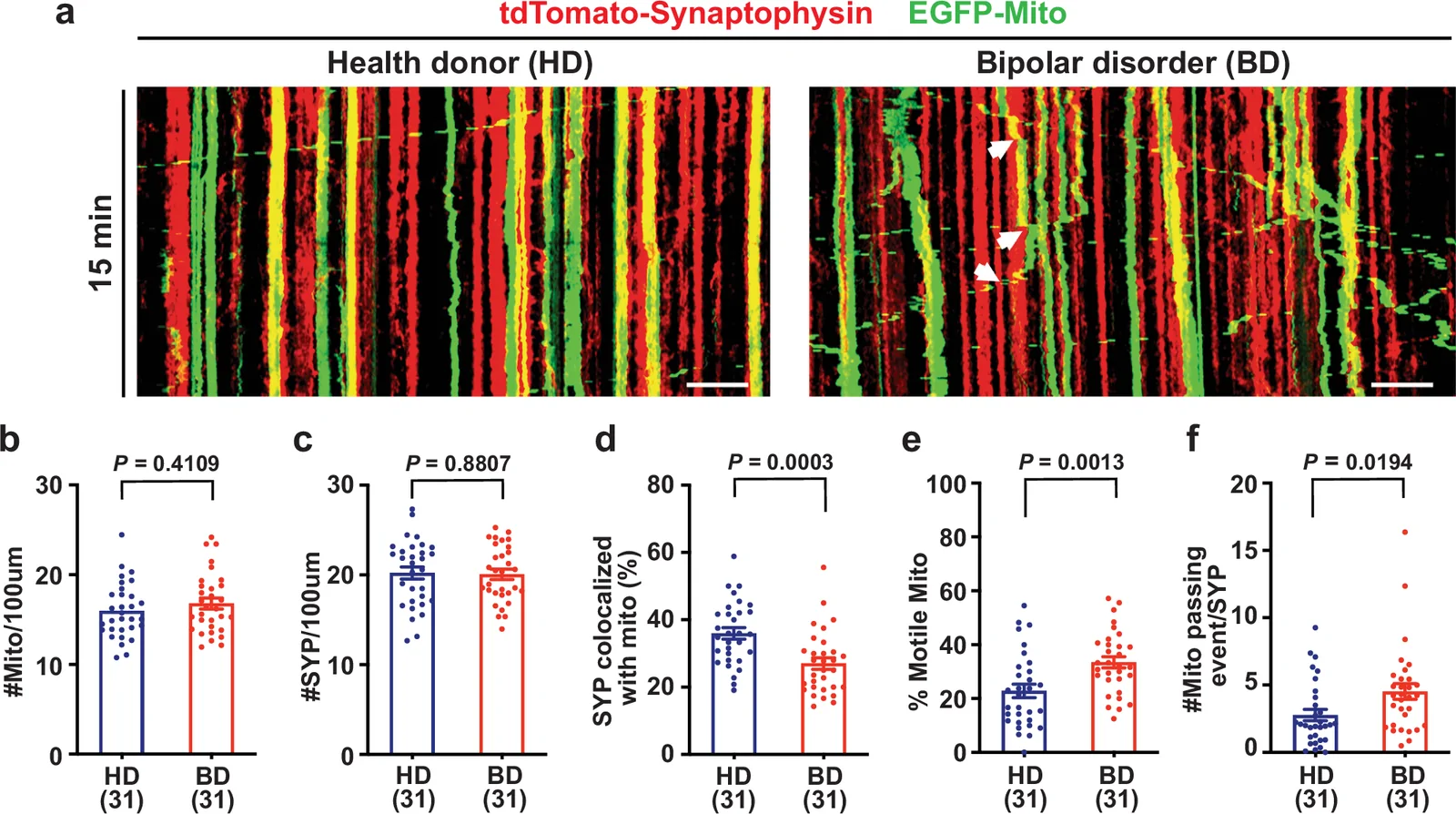
hiPSC-derived neurons from donors with bipolar disorder exhibit increased mitochondrial transit events at presynaptic terminals. Dual-color time-lapse imaging of mitochondrial dynamics at individual presynaptic terminals (**a**). hiPSC-derived cortical neurons from healthy donors (HD) and donors with bipolar disorder were co-transduced at DPI20 with tdTomato–Synaptophysin (red) and EGFP–Mito (green). Live imaging was performed at DPI36-40 (180 frames at 5-s intervals; 15 min total) to quantify mitochondrial transit events at presynaptic terminals. In the kymographs, vertical red lines represent presynaptic terminals, vertical green lines indicate stationary mitochondria, yellow signals denote mitochondria stably retained at presynaptic sites, and diagonal or curved green trajectories indicate motile mitochondria transiting between synapses. A representative motile mitochondrion (white arrows) dynamically enters, transiently pauses within, and subsequently exits a presynaptic terminal before moving to neighboring synapses. Axonal mitochondrial density (*P* = 0.4109; **b**) and presynaptic density (*P* = 0.8807; **c**) were comparable between groups. In contrast, presynaptic mitochondrial occupancy was significantly reduced in neurons from donors with bipolar disorder (*P* = 0.0003; **d**), accompanied by ~50% increase in axonal mitochondrial motility (*P* = 0.0013; **e**). Consistently, mitochondrial transit events per presynaptic terminal during the 15-min recording period increased from 2.77 in neurons from healthy donor to 4.52 in neurons from donors with bipolar disorder (63% increase; *P* = 0.0194; **f**). Data are presented as mean ± s.e.m. from the number of neurons indicated in parentheses across three independent experiments and were analyzed using a two-tailed Mann-Whitney test for individual comparisons (**b**–**f**). Scale bar, 10 µm (**a**). Source data are provided as a Source Data file.

Because hiPSC-derived neurons from donors with bipolar disorder exhibit an approximately 50% increase in axonal mitochondrial motility (Fig. 1h, i), we next examined how this hypermotility influences stable presynaptic mitochondrial occupancy using dual-color live imaging of the presynaptic marker tdTomato-Synaptophysin and the mitochondrial marker EGFP-Mito (Fig. 3a). Axonal mitochondrial density (Fig. 3b) and presynaptic density (Fig. 3c) were comparable between neurons from healthy donors and donors with bipolar disorder. However, neurons from donors with bipolar disorder exhibited reduced presynaptic mitochondrial occupancy in the initial imaging frame (Fig. 3d, *P* = 0.0003), an approximately 50% increase in axonal mitochondrial motility (Fig. 3e, *P* = 0.0013), and a 63% increase in mitochondrial entry-exit transit events at individual presynaptic terminals during a 15-min imaging period (Fig. 3f, *P* = 0.0194). A representative example (Fig. 3a; arrows) shows a motile mitochondrion entering a presynaptic terminal, pausing transiently, and subsequently exiting to pass additional synapses along the axon.

These observations indicate that presynaptic terminals in hiPSC-derived neurons from donors with bipolar disorder experience more frequent mitochondrial entry–exit transitions and reduced stable mitochondrial residency. Such dynamic transients generate a heterogeneous population of presynaptic terminals and dynamically destabilize—rather than uniformly reduce—local ATP supply and Ca^2+^ buffering at individual presynaptic terminals. Accordingly, while mean-level measures of population- and time-averaged release probability or EPSC amplitudes do not capture this distinct phenotype, analyses of pulse-to-pulse and trial-to-trial variability revealed increased fluctuations in synaptic transmission (Fig. 2 and Supplementary Fig. 2). Together, these findings are consistent with increased axonal mitochondrial motility contributing to elevated synaptic variability in hiPSC-derived neurons from donors with bipolar disorder.

### Mouse bipolar disorder-risk neurons exhibit similar synaptoenergetic deficits

To determine whether the impaired presynaptic energy maintenance observed in hiPSC-derived neurons from donors with bipolar disorder is also present in a genetic risk model, we generated AKAP11-deficient mouse cortical neurons using shRNA-mediated knockdown (Supplementary Fig. 3a, b). Protein-truncating variants in AKAP11, which encodes an A-kinase anchoring protein that organizes cAMP-dependent protein kinase A signaling, have been associated with increased risk of bipolar disorder^43^. However, the cellular consequences of AKAP11 deficiency remain incompletely understood. To assess presynaptic energy homeostasis during sustained synaptic activity, cultured cortical neurons were stimulated with the GABA_A_ receptor antagonist picrotoxin (PTX, 100 µM, 30 min). Compared with scramble shRNA controls, AKAP11-deficient neurons (AKAP11-shRNA) exhibited significantly lower presynaptic ATP levels (*P* = 0.0032; Supplementary Fig. 3c, d). AKAP11 deficiency was also associated with reduced presynaptic mitochondrial retention (*P* < 0.0001; Supplementary Fig. 3e, f) and increased axonal mitochondrial motility (*P* < 0.0001; Supplementary Fig. 3g, h). These findings indicate that reduced presynaptic ATP maintenance, decreased presynaptic mitochondrial retention, and increased axonal mitochondrial motility are shared phenotypes of hiPSC-derived neurons from donors with bipolar disorder and AKAP11-deficient mouse neurons. Although AKAP11 regulates multiple neuronal signaling pathways^58^, these results identify a synaptoenergetic phenotype associated with AKAP11 deficiency that parallels the defects observed in hiPSC-derived neurons from donors with bipolar disorder and provide a framework for investigating how AKAP11 dysfunction contributes to synaptic dysfunction in bipolar disorder.

### Lithium restores synaptoenergetics in human and mouse bipolar disorder-relevant neuronal models

**Fig. 4:**
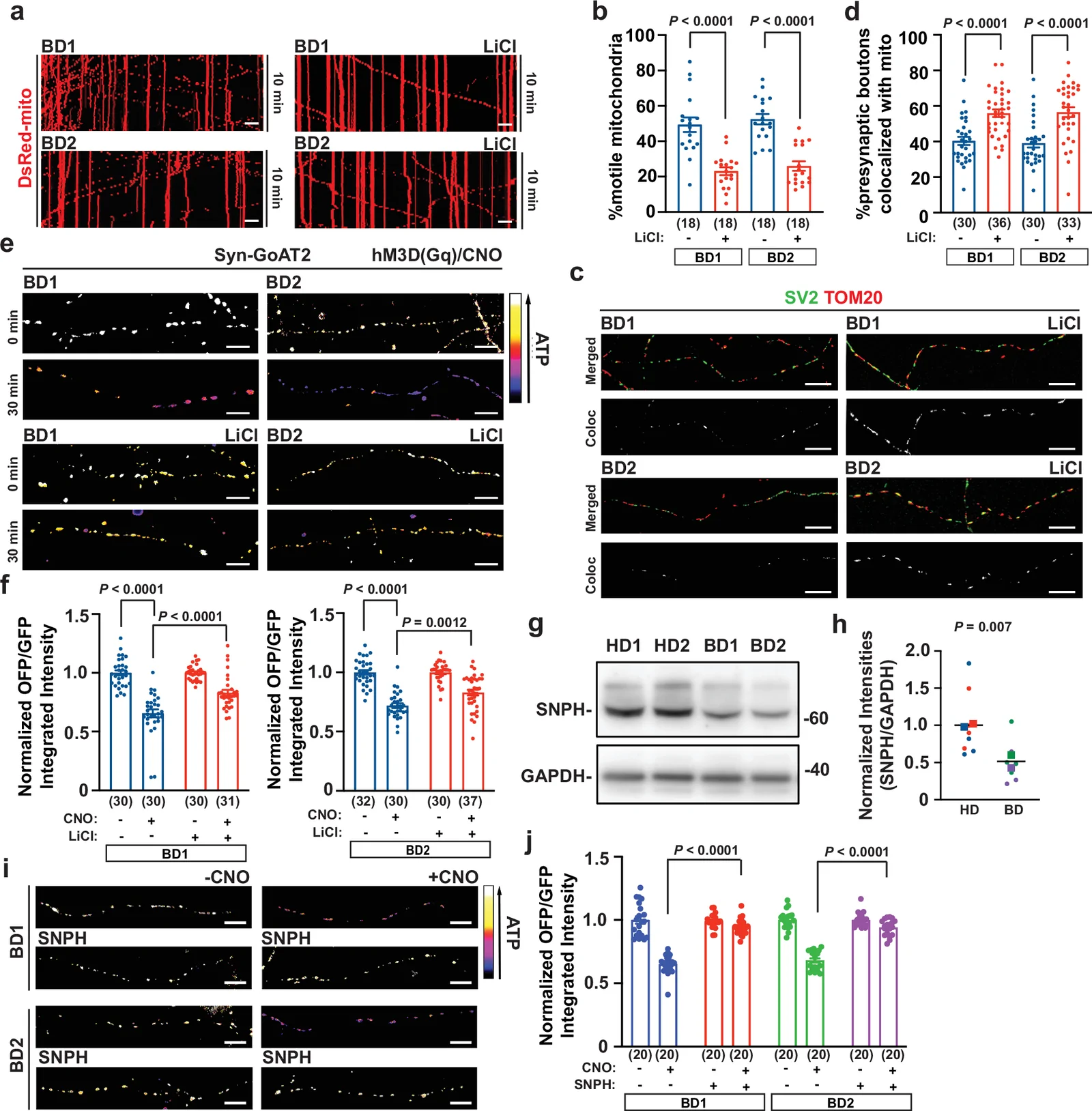
Lithium restores synaptoenergetics in hiPSC-derived neurons from donors with bipolar disorder. **a**, **b** Lithium (LiCl) reduced axonal mitochondrial motility in neurons from donors with bipolar disorder (*P* < 0.0001). Neurons were transfected with DsRed-Mito at DPI14 and treated with DMSO or LiCl (100 µM) from DPI17 to DPI21. Time-lapse imaging was performed for 10 min at DPI21. **c**, **d** LiCl restores presynaptic mitochondrial retention (*P* < 0.0001). Following the same treatment paradigm, neurons were co-immunostained for SV2 and TOM20 at DPI21. Colocalized pixels (white in binary images) indicate presynaptic mitochondria. **e**, **f** LiCl attenuates activity-induced presynaptic ATP depletion (*P* < 0.0001). hiPSC-derived neurons expressing hM3D(Gq) were transfected with Syn-GoATeam2 at DPI14 and treated with DMSO or LiCl (100 µM) from DPI17 to DPI21. Live imaging was performed before and after 30-min stimulation with CNO (10 µM). The OFP/GFP ratio indicates relative presynaptic ATP levels. The pseudocolor bar scale represents fluorescence intensity in arbitrary units (AU), with blue indicating low ATP and red indicating high ATP. **g**, **h** Reduced SNPH expression in hiPSC-derived cortical neurons from donors with bipolar disorder. **g** Immunoblots of neuronal lysates (5 µg) collected at DPI21. **h** Quantification of SNPH protein levels normalized to GAPDH and expressed relative to healthy donor neurons (*P* = 0.007). Comparison was made between 8 HD and 8 BD biological replicates (4 from each cell type). **i**, **j** Re-expression of SNPH rescues activity-induced presynaptic ATP deficits in hiPSC-derived cortical neurons from donors with bipolar disorder. Neurons expressing hM3D(Gq) together with HA or HA-SNPH were transfected with Syn-GoATeam2 at DPI14. Live imaging was performed at DPI21 before and after stimulation with CNO (10 μM). The OFP/GFP ratio indicates relative presynaptic ATP levels. Data were presented as mean ± s.e.m. from the number of neurons indicated in parentheses or within bar graphs across three independent experiments and were analyzed using one-way ANOVA with Tukey’s post-hoc test (**b**, **d**, **f**, **j**) or a two-tailed Mann-Whitney test (**h**). Scale bars: 10 µm. Source data are provided as a Source Data file.

Lithium is a first-line mood stabilizer for the treatment of acute mania and prevention of recurrent mood episodes in bipolar disorder^20^. Although its clinical efficacy is well established, the cellular mechanisms underlying its therapeutic effects remain incompletely understood^59^. Recent pharmacogenetic studies have highlighted patient-specific variability in lithium responses, underscoring the importance of defining its mechanistic actions in disease-relevant neuronal models^20^. We therefore examined whether lithium rescues activity-induced synaptoenergetic deficits in hiPSC-derived neurons from donors with bipolar disorder and AKAP11-deficient mouse neurons. Human neurons and AKAP11-deficient mouse neurons were treated with lithium chloride (LiCl; 100 μM) for 4 days. In hiPSC-derived neurons from donors with bipolar disorder, LiCl significantly reduced axonal mitochondrial motility (BD1 vs. BD1 + LiCl, *P* < 0.0001; BD2 vs. BD2 + LiCl, *P* < 0.0001; Fig. 4a, b). Consistent with reduced mitochondrial motility, LiCl increased presynaptic mitochondrial retention, as determined by SV2 and TOM20 colocalization (BD1 vs. BD1 + LiCl, *P* < 0.0001; BD2 vs. BD2 + LiCl, *P* < 0.0001; Fig. 4c, d). Using the presynaptic ATP sensor Syn-GoATeam2 together with hM3D(Gq)/CNO stimulation, we further found that LiCl significantly attenuated activity-induced presynaptic ATP depletion after 30 min of stimulation (BD1 vs. BD1 + LiCl, *P* < 0.0001; BD2 vs. BD2 + LiCl, *P* = 0.0112; Fig. 4e, f).

Similar effects were observed in AKAP11-deficient mouse neurons. LiCl significantly reduced axonal mitochondrial motility (*P* < 0.0001; Supplementary Fig. 3i, j), increased presynaptic mitochondrial retention (*P* < 0.0001; Supplementary Fig. 3k, l), and attenuated activity-induced presynaptic ATP depletion (*P* < 0.0001; Supplementary Fig. 3m, n). Together, these findings indicate that lithium improves presynaptic mitochondrial retention and ATP maintenance during sustained neuronal activity in both iPSC-derived human neurons from donors with bipolar disorder and AKAP11-deficient mouse neurons. These results are consistent with enhanced presynaptic mitochondrial support contributing to the cellular actions of lithium in bipolar disorder-relevant neuronal models.

### Mitochondrial bioenergetics remain intact in bipolar disorder-relevant neurons

To determine whether the activity-induced presynaptic energy deficits observed in bipolar disorder-relevant neurons result from intrinsic mitochondrial dysfunction, we assessed mitochondrial bioenergetic capacity. First, mitochondrial ATP levels were measured using the mitochondria-targeted ATP sensor mito-GoATeam2 (mito-GoATeam2). Live-cell imaging revealed no significant differences in axonal mitochondrial ATP levels between hiPSC-derived neurons from healthy donors and donors with bipolar disorder (HD1 vs. BD1, *P* = 0.8904; HD1 vs. BD2, *P* = 0.1366; HD2 vs. BD1, *P* = 0.8444; HD2 vs. BD2, *P* = 0.1089) (Supplementary Fig. 4a, b). We next assessed mitochondrial membrane potential (Δψ_m_) using two independent Δψ_m_-sensitive dyes, MitoTracker Orange CMTMRos and tetramethylrhodamine ethyl ester (TMRE). Neither assay detected significant differences between neurons from healthy donors and donors with bipolar disorder (Supplementary Fig. 4c–f). Seahorse extracellular flux analysis likewise showed comparable basal respiration, maximal respiration, spare respiratory capacity, and ATP-linked respiration between groups (Supplementary Fig. 4g–k).

We next examined mitochondrial function in AKAP11-deficient mouse cortical neurons. Neither mitochondrial membrane potential, measured with MitoTracker Orange CMTMRos (*P* = 0.5871; Supplementary Fig. 4l, m), nor mitochondrial ATP levels measured with mito-GoATeam2 (*P* = 0.9740; Supplementary Fig. 4n, o) differed between AKAP11-deficient and control neurons. In addition, overall mitochondrial density and subcellular distribution in axons and somatodendritic compartments were comparable between hiPSC-derived neurons from healthy donors and donors with bipolar disorder (Supplementary Fig. 5). These findings indicate that the presynaptic energy deficits observed in human and mouse bipolar disorder-relevant neuronal models are not associated with detectable changes in intrinsic mitochondrial bioenergetic capacity or mitochondrial abundance. Instead, they are consistent with impaired mitochondrial retention at presynaptic terminals contributing to reduced ATP maintenance during sustained neuronal activity. This distinction is notable because widespread mitochondrial dysfunction would be expected to compromise neuronal energy metabolism broadly, whereas the changes observed here are restricted to presynaptic energy homeostasis.

### SNPH deficiency contributes to presynaptic energy deficits in hiPSC-derived neurons from donors with bipolar disorder

Syntaphilin (SNPH) is an axonal mitochondrial anchoring protein that retains mitochondria at presynaptic terminals. Loss of SNPH in mouse cortical neurons increases axonal mitochondrial motility, reduces presynaptic mitochondrial retention, and impairs presynaptic ATP maintenance during sustained neuronal activity^9,10,12^. These phenotypes resemble those observed in hiPSC-derived neurons from donors with bipolar disorder, suggesting a common synaptoenergetic mechanism. Consistent with this possibility, an unbiased proteomic analysis of synapse-enriched fractions from the prefrontal cortex of 35 individuals with bipolar disorder and 35 control subjects reported a 21% reduction in SNPH protein abundance in bipolar disorder brains^60^. We therefore examined SNPH expression in hiPSC-derived human cortical neurons. Immunoblot analysis revealed an average 51.42% reduction in SNPH protein levels in neurons from donors with bipolar disorder compared with neurons from healthy donors (*P* = 0.0070) (Fig. 4g, h). This reduction in SNPH expression is consistent with the increased axonal mitochondrial motility, reduced presynaptic mitochondrial retention, and activity-induced presynaptic ATP deficits observed in these neurons.

To determine whether restoring SNPH expression affects presynaptic energy homeostasis, we re-expressed SNPH in human BD1 and BD2 neurons, which exhibit approximately 50% lower endogenous SNPH levels. Elevated SNPH expression significantly increased presynaptic ATP levels during sustained neuronal activity in both hiPSC-derived neuronal lines (Fig. 4i, j). These findings indicate that reduced SNPH expression contributes to impaired presynaptic ATP maintenance in hiPSC-derived BD neuronal models and that restoring SNPH expression is required to maintain presynaptic energy homeostasis under sustained neuronal activity.

### *Snph* knockout mice recapitulate mania-like behavioral phenotypes

**Fig. 5:**
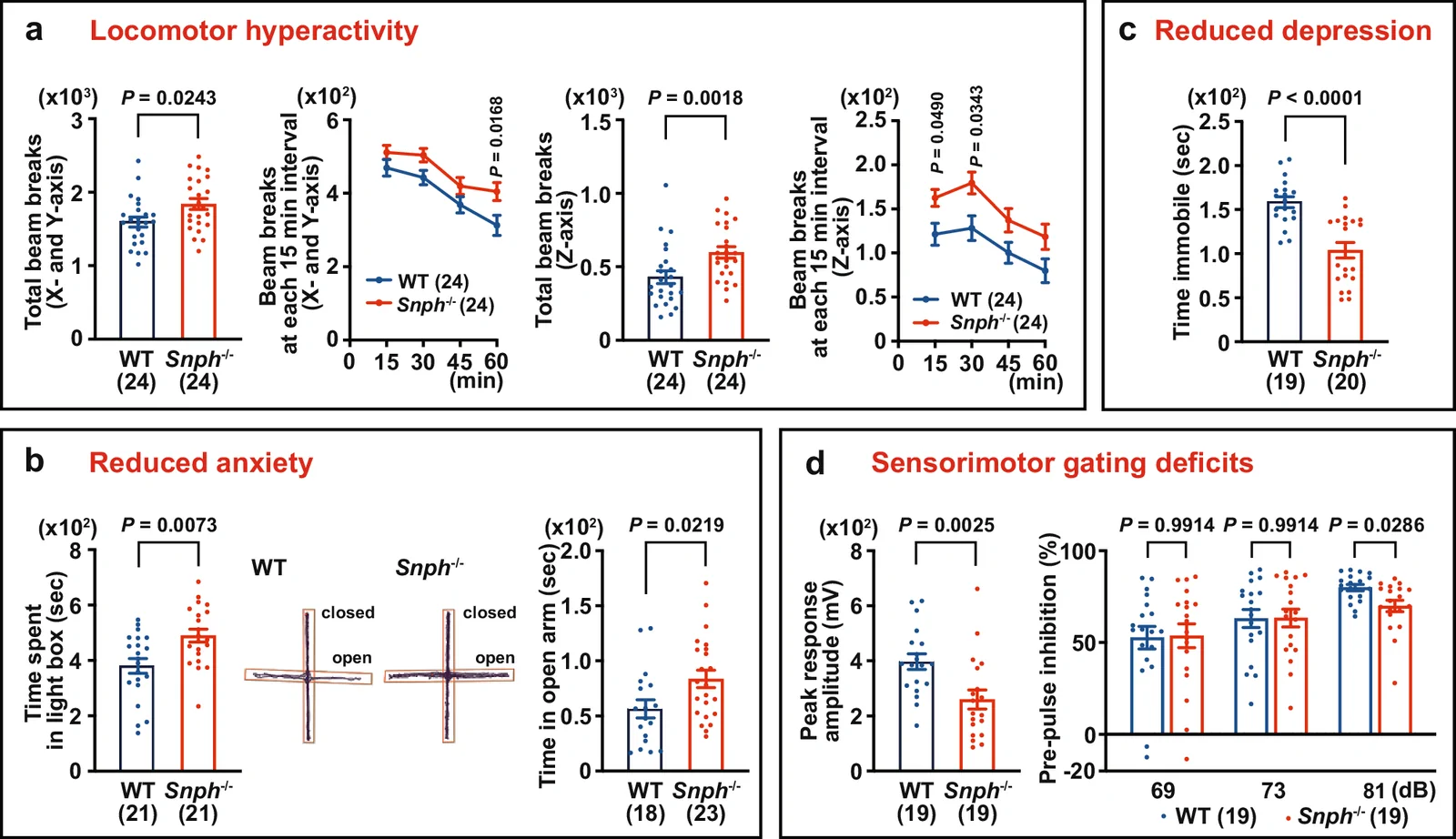
*Snph*⁻^/^⁻ mice exhibit mania-like behavioral phenotypes. **a** *Snph*⁻^/^⁻ mice display locomotor hyperactivity. Twelve- to fourteen-week-old WT (*n* = 24) and *Snph*⁻^/^⁻ mice (*n* = 24) were assessed in an open-field activity chamber. Horizontal locomotion was quantified by cumulative X- and Y-axis beam breaks over 60 min and across 15-min intervals. Vertical activity (rearing) was quantified by Z-axis beam breaks. *Snph*⁻^/^⁻ mice exhibited significantly increased ambulatory activity (60 min: *P* = 0.0243; time course: two-way ANOVA *F*_*1, 46*_ = 6.099, *P* = 0.0173) and rearing behavior (60 min: *P* = 0.0018; time course: two-way ANOVA *F*_*1,46*_ = 8.359, *P* = 0.0058) compared with WT mice. **b** *Snph*⁻^/^⁻ mice exhibit reduced anxiety-like behavior. Anxiety-related behavior was assessed using the Light–Dark Box (LDB) and Elevated Plus Maze (EPM). Compared with WT mice, *Snph*⁻^/^⁻ mice spent significantly more time in the light compartment of the LDB (WT: *n* = 21; *Snph*⁻^/^⁻: *n* = 21; *P* = 0.0073) and in the open arms of the EPM (WT: *n* = 18; *Snph*⁻^/^⁻: *n* = 23; *P* = 0.0219). **c** *Snph*⁻^/^⁻ mice exhibit reduced depression-like behavior in the Forced Swim Test (FST). Compared with WT mice (*n* = 19), *Snph*⁻^/^⁻ mice (*n* = 20) displayed significantly reduced immobility time (*P* < 0.0001). **d** *Snph*⁻^/^⁻ mice display impaired sensorimotor gating. Acoustic startle response and pre-pulse inhibition (PPI) tests were measured. Compared to WT mice (*n* = 19), *Snph*⁻^/^⁻ mice (*n* = 19) exhibited reduced startle response at 120 dB (*P* = 0.0025) and impaired PPI at a pre-pulse intensity of 81 dB (*P* = 0.0286). Data are presented as mean ± s.e.m. from the number of individual mice indicated in parentheses in each group across three independent batches of behavioral experiments. Each animal represents one biological replicate. Statistical analyses were performed using two-tailed Mann-Whitney test for pairwise comparisons (**a**–**d**) or two-way ANOVA with genotype and treatment as independent variables, followed by Holm–Šidák multiple-comparison tests for interactions (**a**). Source data are provided as a Source Data file.

Because *Snph* knockout (KO) mice exhibit increased axonal mitochondrial motility and reduced presynaptic mitochondrial retention, we examined whether these synaptoenergetic defects are associated with bipolar disorder-related behaviors. Age-matched (12–14-week-old) male *Snph* KO mice and wild-type (WT) littermates were subjected to a behavioral test battery. In the open-field assay, *Snph* KO mice exhibited significantly greater locomotor activity than WT mice, measured by cumulative X- and Y-axis beam breaks over 60 min (*P* = 0.0243; Fig. 5a). This increase was most pronounced during the fourth 15-min interval (two-way ANOVA, genotype: *F*_*1,46*_ = 6.099, *P* = 0.0173; 4th interval: *P* = 0.0168). *Snph* KO mice also displayed increased rearing activity, reflected by a greater number of Z-axis beam breaks over 60 min (*P* = 0.0018), with significant differences during the first and second 15-min intervals (two-way ANOVA, genotype: *F*_*1,46*_ = 8.359, *P* = 0.0058; first interval: *P* = 0.0490; second interval: *P* = 0.0343). These results indicate that *Snph* KO mice exhibit locomotor hyperactivity and increased exploratory behavior, phenotypes consistent with psychomotor agitation—a hallmark feature of mania episodes in BD patients^61^.

We next assessed anxiety- and depression-related behaviors^62,63^. In both the light-dark box (LDB) and elevated plus maze (EPM), *Snph* KO mice spent significantly more time in the light compartment (*P* = 0.0073) and open arms (*P* = 0.0219), respectively, than WT littermates (Fig. 5b). In the forced swim test, *Snph* KO mice exhibited reduced immobility time compared with WT mice (*P* < 0.0001; Fig. 5c). Sensorimotor gating, which enables the filtering out of irrelevant sensory information, was assessed using acoustic startle response and prepulse inhibition (PPI)^64–66^. Compared with WT mice, *Snph* KO mice exhibited reduced startle responses to a 120-dB stimulus (*P* = 0.0025) and impaired PPI at an 81-dB prepulse intensity (*P* = 0.0286; Fig. 5d), indicating altered sensorimotor gating. Together, these findings demonstrate a behavioral profile characterized by increased psychomotor activity, reduced anxiety- and depression-like behaviors, and impaired sensorimotor gating, consistent with mania-like behavioral phenotypes.

To determine whether partial reduction of SNPH is sufficient to produce similar behavioral changes, we compared WT, heterozygous (*Snph*^+/–^), and homozygous (*Snph*^–/–^) mice. *Snph*^+/–^ mice exhibited locomotor hyperactivity (XY-axis, *P* = 0.0026), increased rearing activity (Z-axis, *P* = 0.002), and reduced immobility in the forced swim test (*P* = 0.0086), but did not show significant changes in anxiety-related behaviors (Supplementary Fig. 6). Because *Snph*^+/–^ mice express approximately 50% of normal SNPH levels, comparable to the reduction observed in hiPSC-derived neurons from donors with bipolar disorder, these findings indicate that partial loss of SNPH is associated with key mania-like behavioral phenotypes. Together, *Snph*-deficient mice exhibit behavioral alterations that parallel several features associated with manic episodes in bipolar disorder, supporting their use for investigating the relationship between presynaptic mitochondrial retention, synaptoenergetic homeostasis, and mood-related behaviors.

### Lithium reverses mania-like behavioral phenotypes in *Snph* KO mice

**Fig. 6:**
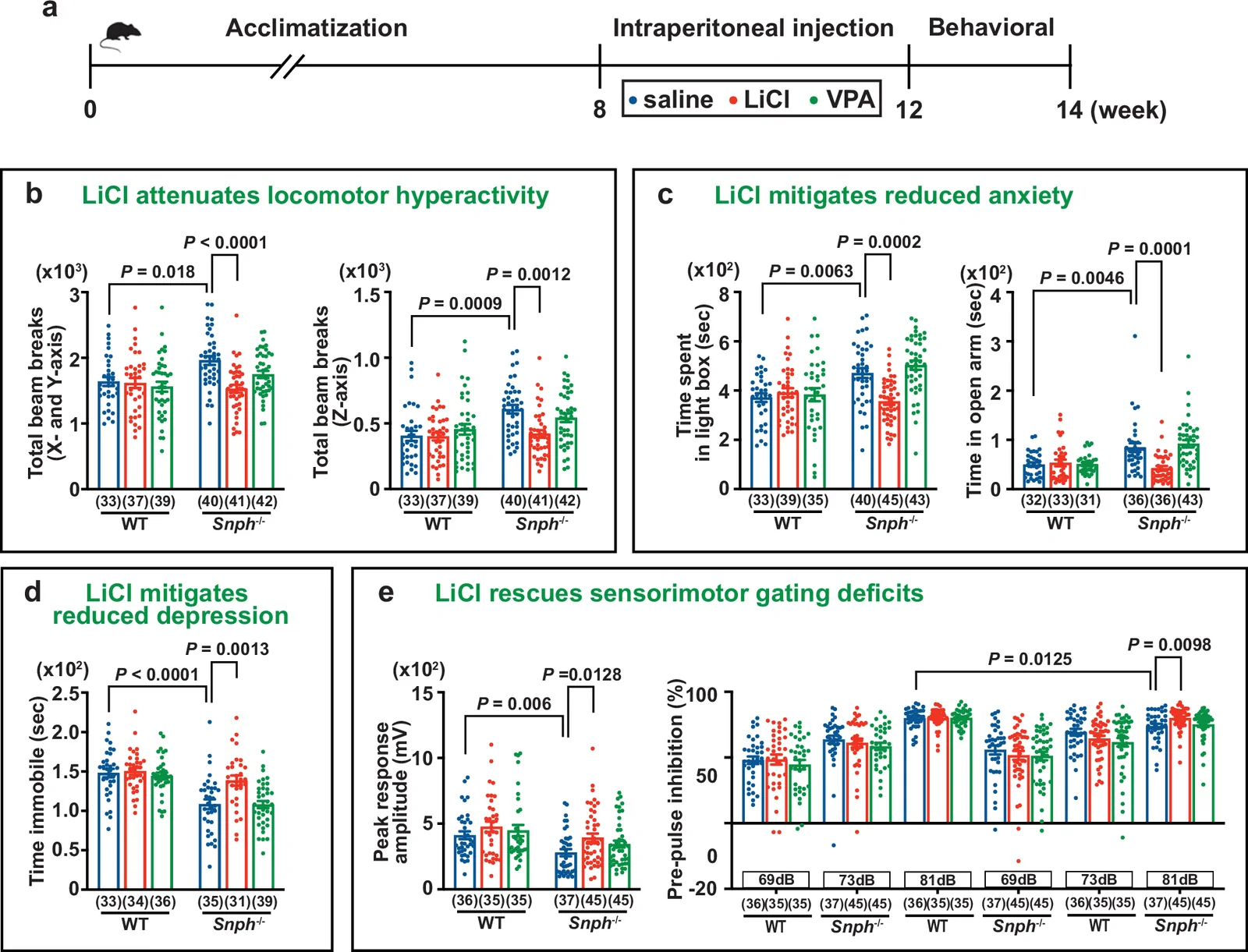
Lithium reverses mania-like behavioral phenotypes in *Snph*⁻^/^⁻ mice. **a** Experimental timeline. WT and *Snph*⁻^/^⁻ mice received daily intraperitoneal injections of saline, lithium chloride (LiCl; 90 mg/kg), or valproic acid (VPA; 400 mg/kg) from 8 to 12 weeks of age. Behavioral testing was performed between 12 and 16 weeks of age. **b** LiCl, but not VPA, attenuates locomotor hyperactivity in *Snph*⁻^/^⁻ mice. LiCl significantly reduced cumulative horizontal locomotor activity (two-way ANOVA: *F*_*2,226*_ = 4.734, *P* = 0.0097) and rearing behavior (two-way ANOVA: *F*_*2, 226*_ = 3.575, *P* = 0.0296), whereas VPA had no significant effect. **c** LiCl, but not VPA, rescues reduced anxiety-like behavior in *Snph*⁻^/^⁻ mice. LiCl significantly decreased the time spent in the light compartment of the Light–Dark Box (LDB) (two-way ANOVA: *F*_*2, 229*_ = 10.01, *P* < 0.0001) and in the open arms of the EPM (two-way ANOVA: *F*_*2, 205*_ = 9.380, *P* = 0.0001), indicating partial normalization of the anxiolytic phenotype. **d** LiCl, but not VPA, attenuates reduced depression-like behavior. In the Forced Swim Test (FST), LiCl significantly increased immobility time in *Snph*⁻^/^⁻ mice (two-way ANOVA: *F*_*2, 202*_ = 4.153, *P* = 0.0171), whereas VPA produced no significant effect. **e** LiCl, but not VPA, rescues sensorimotor gating deficits. LiCl restored the reduced acoustic startle response at 120 dB (two-way ANOVA: *F*_*2, 227*_ = 3.596, *P* = 0.0290) and improved prepulse inhibition (PPI) at a prepulse intensity of 81 dB (saline vs LiCl, *P* = 0.0098) in *Snph*⁻^/^⁻ mice, whereas VPA had no significant effect. The number of age-matched male WT and *Snph*⁻^/^⁻ mice in each treatment group is indicated in parentheses across three independent batches of behavioral experiments. Each animal represents one biological replicate. Data are presented as mean ± s.e.m. Statistical analyses were performed using two-way ANOVA with genotype and treatment as independent variables, followed by Holm–Šidák post-hoc tests for interactions (**b**–**e**). Source data are provided as a Source Data file.

To determine whether restoration of synaptoenergetic homeostasis is associated with improved behavioral outcomes, *Snph* KO mice received chronic lithium chloride (LiCl) treatment via intraperitoneal injection, followed by behavioral testing (Fig. 6a). Valproic acid (VPA), a clinically approved mood stabilizer by primarily enhancing GABAergic signaling^67,68^, was included for comparison. Brain and serum lithium concentrations measured by inductively coupled plasma mass spectrometry (ICP-MS) were within the therapeutic range reported in humans (0.6–1.5 mmol/L)[69,^69,70^ (Supplementary Fig. 7a, b). Serum VPA concentrations, measured by low-resolution electrospray ionization mass spectrometry (LRESIMS), were also within the therapeutic range (50–100 mg/L)^71^ (Supplementary Fig. 7c). Neither LiCl nor VPA altered behavioral performance in WT mice (Fig. 6).

We next examined the effects of LiCl and VPA on behavioral phenotypes in *Snph* KO mice. In the open-field assay, LiCl significantly reduced hyperlocomotion (XY-axis, KO + saline vs. KO + LiCl, *P* < 0.0001) and rearing activity (Z-axis, KO + saline vs. KO + LiCl*, P* = 0.0012), whereas VPA had no significant effect (Fig. 6b). LiCl also normalized anxiety-related behaviors in both the light-dark box (LDB, KO + saline vs. KO + LiCl*, P* = 0.0002) and elevated plus maze (EPM, KO + saline vs. KO + LiCl*, P* = 0.0001; Fig. 6c), and restored immobility time in the forced swim test (FST, KO + saline vs. KO + LiCl*, P* = 0.0013; Fig. 6d). In contrast, VPA did not significantly affect anxiety- or depression-like behaviors (LDB, KO + saline vs. KO + VPA, *P* = 0.8272; EPM, KO + saline vs. KO + VPA*, P* = 0.9482; FST, KO + saline vs. KO + VPA*, P* > 0.9999). LiCl further improved acoustic startle responses (KO + saline vs. KO + LiCl*, P* = 0.0128) and prepulse inhibition (PPI) at an 81-dB prepulse intensity (KO + saline vs. KO + LiCl*, P* = 0.0098), whereas VPA had no significant effect on either measure (Fig. 6e).

Together, these findings indicate that chronic lithium treatment reverses mania-like behavioral phenotypes in *Snph* KO mice, including hyperlocomotion, reduced anxiety- and depression-like behaviors, and impaired sensorimotor gating. These behavioral improvements are consistent with the restoration of presynaptic mitochondrial retention and synaptoenergetic homeostasis observed in lithium-treated neuronal models.

### LiCl restores presynaptic energetics and Ca^2+^ buffering in *Snph* KO neurons

**Fig. 7:**
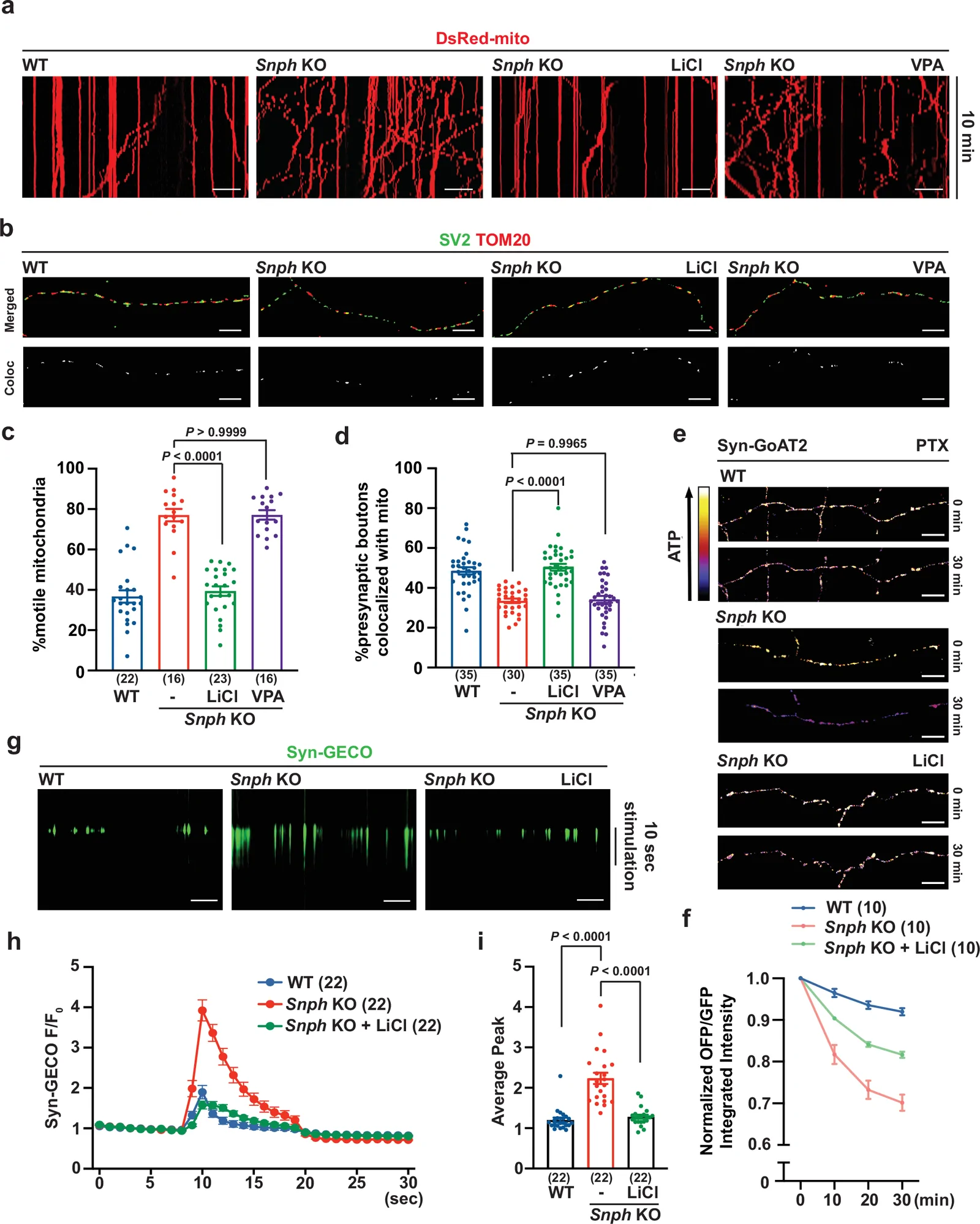
Lithium restores presynaptic mitochondrial retention and synaptoenergetics in *Snph* KO neurons. **a**–**d** LiCl rescues presynaptic mitochondrial retention in *Snph* KO cortical neurons. Representative time-lapse images of DsRed-Mito (**a**) and immunostaining for TOM20 and SV2 (**b**). Colocalized pixels (white in binary images) indicate presynaptic mitochondria. Quantification shows that LiCl, but not valproic acid (VPA), significantly reduced axonal mitochondrial motility (*P* < 0.0001, **c**) and restored presynaptic mitochondrial retention (*P* < 0.0001, **d**) in *Snph* KO neurons. **e**, **f** LiCl attenuates activity-induced presynaptic ATP depletion. Cortical neurons expressing the presynaptic ATP sensor Syn-GoATeam2 were imaged before and after stimulation with PTX (100 µM). Representative ATP heatmaps are shown in (**e**). Quantification (**f**) revealed significant effects of genotype/treatment (*F*₂_,_₂₇ = 50.78, *P* < 0.0001) and genotype/treatment × time interaction (*F*₆_,_₈₁ = 41.08, *P* < 0.0001). LiCl significantly attenuated ATP decline in *Snph* KO neurons at 10 min (*P* = 0.0102), 20 min (*P* = 0.0018), and 30 min (*P* = 0.0005). The pseudocolor bar scale represents fluorescence intensity in arbitrary units (AU), with blue indicating low ATP and red indicating high ATP. **g**–**i** LiCl restores presynaptic Ca^2+^ clearance in *Snph* KO neurons. Representative Syn-GECO kymographs (**g**) and normalized presynaptic Ca^2+^ transients (**h**) following a 100-Hz stimulation train. Vertical green lines represent presynaptic [Ca^2+^]_i_ transients during stimulation train. LiCl treatment significantly restored presynaptic [Ca^2+^]_i_ clearance in *Snph* KO neurons during intense activity (*P* < 0.0001, **i**). Data are presented as mean ± s.e.m. from the number of neurons indicated in the graphs or parentheses across three independent experiments. Statistical analyses were performed using one-way ANOVA with Tukey’s multiple-comparison test (**c**, **d**, **i**) or two-way ANOVA followed by Tukey’s multiple-comparison test (**f**). Scale bars: 20 μm (**a**, **g**) and 10 μm (**b**, **e**). Source data are provided as a Source Data file.

To determine whether lithium restores presynaptic function in the absence of SNPH, we examined the effects of chronic LiCl treatment (100 μM, 4 days) on axonal mitochondrial motility and presynaptic mitochondrial retention in WT and *Snph* KO cortical neurons. LiCl significantly reduced axonal mitochondrial motility in *Snph* KO neurons from 77.03 ± 3.08% to 38.93 ± 2.41%, comparable to WT neurons (KO vs. KO + LiCl, *P* < 0.0001; KO + LiCl vs. WT, *P* = 0.9453; Fig. 7a, c). Consistent with this change, LiCl increased presynaptic mitochondrial retention from 33.48 ± 1.01 to 50.35 ± 1.71% (*P* < 0.0001), restoring levels comparable to WT neurons (*P* = 0.8619; Fig. 7b, d). In contrast, valproic acid (VPA; 1 mM, 4 days) did not significantly affect axonal mitochondrial motility (*P* > 0.9999) or presynaptic mitochondrial retention (*P* = 0.9965). These findings indicate that lithium restores presynaptic mitochondrial retention in *Snph* KO neurons despite the absence of SNPH.

We next examined whether LiCl improves presynaptic ATP maintenance during sustained neuronal activity. In WT neurons, picrotoxin (PTX) induced a modest decline in presynaptic ATP levels, whereas *Snph* KO neurons exhibited a significantly greater reduction at 10 min (*P* = 0.0002), 20 min (*P* < 0.0001), and 30 min (*P* < 0.0001; Fig. 7e, f). LiCl significantly attenuated ATP depletion in *Snph* KO neurons at 10 min (*P* = 0.0102), 20 min (*P* = 0.0018), and 30 min (*P* = 0.0005). Because presynaptic mitochondria also regulate intracellular Ca^2+^ dynamics which are essential for short-term synaptic plasticity^54,72^, we monitored presynaptic Ca^2+^ transients using the synaptic vesicle-targeted Ca^2+^ indicator Syn-GECO during a 100-Hz stimulation train. *Snph* KO neurons exhibited slower presynaptic Ca^2+^ clearance than WT neurons (*P* = 0.0001; Fig. 7g–i). LiCl significantly improved Ca^2+^ clearance in *Snph* KO neurons (*P* < 0.0001). Together, these findings indicate that lithium improves presynaptic mitochondrial retention, ATP maintenance, and Ca^2+^ buffering in *Snph* KO neurons. These cellular changes are consistent with the behavioral improvements observed in lithium-treated *Snph* KO mice and support an association between impaired presynaptic mitochondrial retention, activity-induced synaptoenergetic deficits, and mania-like behavioral phenotypes.

### Lithium normalizes synaptic variability in *Snph* KO neurons

**Fig. 8:**
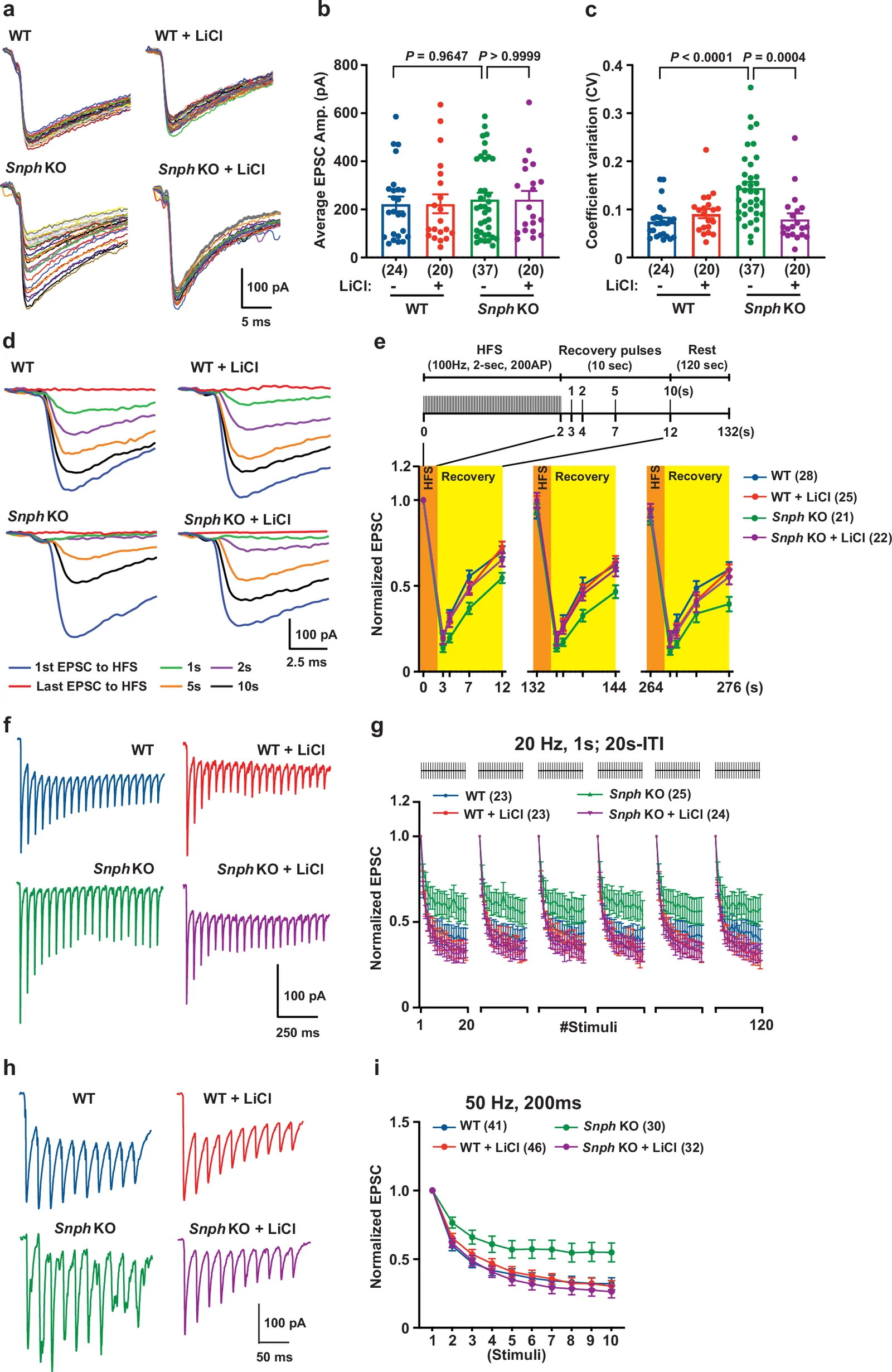
LiCl reduces synaptic variability and restores synaptic efficacy in *Snph* KO neurons. **a**–**c** LiCl reduces pulse-to-pulse variability of evoked EPSC in *Snph* KO cortical neurons. **a** Representative traces of 30 consecutive EPSC sweeps recorded at 0.05-Hz from WT and *Snph* KO neurons with or without LiCl treatment. **b** Mean EPSC peak amplitudes for each neuron and averaged across groups (one-way ANOVA: *F*_*3,97*_ = 0.1227, *P* = 0.9465). **c** Coefficient of variation (CV) of EPSC amplitudes calculated from 30 sweeps per neuron. LiCl significantly reduced synaptic variability in *Snph* KO neurons (one-way ANOVA: *F*_*3,97*_ = 10.01, *P* < 0.0001). **d**, **e** LiCl accelerates recovery of synaptic transmission following high-frequency stimulation (HFS) in *Snph* KO cortical neurons. **d** Representative EPSC traces recorded before, during, and after a 100-Hz stimulation train. **e** Recovery protocol (total 132 s) includes a 2-s 100-Hz train, a 10-s recovery phase with four stimuli, and a 120-sec rest phase. EPSC amplitudes were normalized to the first response of the initial HFS train. Two-way ANOVA revealed significant effects of genotype/treatment (*F*_*3,92*_ = 2.784, *P* = 0.0452) and genotype/treatment x stimulus interaction (*F*_*42,1288*_ = 1.837, *P* = 0.001). **f**–**i** LiCl restores short-term synaptic depression during repetitive stimulation in *Snph* KO neurons. Representative EPSC traces recorded at 20 Hz for 1 s (**f**) and 50 Hz for 200 ms (**h**). Normalized EPSC amplitudes are plotted relative to the first EPSC at 20-Hz (**g**) and 50-Hz (**i**). Two-way ANOVA revealed significant genotype/treatment effects (20 Hz: *F*_*3,91*_ = 3.466, *P* = 0.019; 50 Hz: *F*_*3,145*_ = 5.155, *P* = 0.002) and genotype/treatment x stimulus interaction (20 Hz: *F*_*357,10829*_ = 1.953, *P* < 0.0001; 50 Hz: *F*_*27,1305*_ = 4.314, *P* < 0.0001). Data are presented as mean ± s.e.m. from the number of neuron pairs indicated in parentheses across three independent experiments. Statistical analyses were performed using one-way ANOVA with Tukey’s post-hoc test (**b**, **c**) or two-way ANOVA with genotype and stimulus as independent variables followed by Tukey’s post-hoc test (**e**, **g**, **i**). Source data are provided as a Source Data file.

To determine whether lithium reverses synaptic variability in *Snph* KO neurons, we performed dual whole-cell patch-clamp recordings during 0.05-Hz stimulation. Although the mean peak amplitude of 30 consecutive EPSCs did not differ between WT and *Snph* KO neurons (Fig. 8a, b), *Snph* KO neurons exhibited significantly greater pulse-to-pulse variability in EPSC amplitudes, reflected by an increased coefficient of variation (CV; *P* < 0.0001; Fig. 8c). LiCl treatment (100 μM, 4 days) significantly reduced this variability (*P* = 0.0004), restoring CV values to levels comparable to WT neurons (*P* = 0.9219). These findings are consistent with improved presynaptic mitochondrial retention contributing to more stable synaptic transmission.

Presynaptic mitochondria are also required for synaptic recovery following sustained activity^14^. To determine whether lithium improves synaptic recovery, we applied a high-frequency stimulation (HFS)-recovery protocol consisting of a 2-s, 100-Hz stimulation train, followed by a recovery phase in which four stimuli were delivered over a 10-s interval, repeated after a 120-s rest phase. *Snph* KO neurons exhibited slower recovery than WT neurons (two-way ANOVA, genotype, *F*_*1,47*_ = 7.463, *P* = 0.0088; Fig. 8d, e). LiCl significantly improved recovery kinetics (two-way ANOVA, genotype/treatment x stimulus: *F*_*42,1288*_ = 1.837, *P* = 0.001), restoring recovery to WT levels. We next examined whether lithium restores short-term synaptic plasticity during repetitive stimulation. Following LiCl treatment, *Snph* KO neurons exhibited short-term depression profiles comparable to those of WT neurons (Fig. 8f–i). Two-way ANOVA revealed significant effects of genotype/treatment (20 Hz: *F*_*3,91*_ = 3.466, *P* = 0.0194; 50 Hz: *F*_*3,145*_ = 5.155, *P* = 0.002) and genotype/treatment x stimulus interactions (20 Hz: *F*_*57,1729*_ = 1.953, *P* < 0.0001; 50 Hz: *F*_*27,1305*_ = 4.314, *P* < 0.0001). These findings indicate that lithium restores synaptic recovery and short-term synaptic plasticity in *Snph* KO neurons, consistent with improved presynaptic mitochondrial retention, ATP maintenance, and Ca^2+^ buffering.

### Lithium restores presynaptic mitochondrial retention through the Na^+^/Ca^2+^/Li^+^ Exchanger (NCLX)

Because lithium restored presynaptic mitochondrial retention in *Snph* KO neurons despite the absence of SNPH, we investigated the underlying mechanism. Mitochondrial motility is regulated by Miro1, a mitochondrial outer membrane motor adaptor that arrests mitochondrial movement in response to local Ca^2+^ signals^73–75^. This Ca^2+^-dependent pause is normally stabilized by SNPH-mediated mitochondrial anchoring^10^. In the SNPH deficiency, as observed in *Snph* KO neurons and hiPSC-derived neurons from donors with bipolar disorder, sustained Miro1-Ca^2+^ signaling may provide an alternative mechanism for maintaining presynaptic mitochondrial retention.

**Fig. 9:**
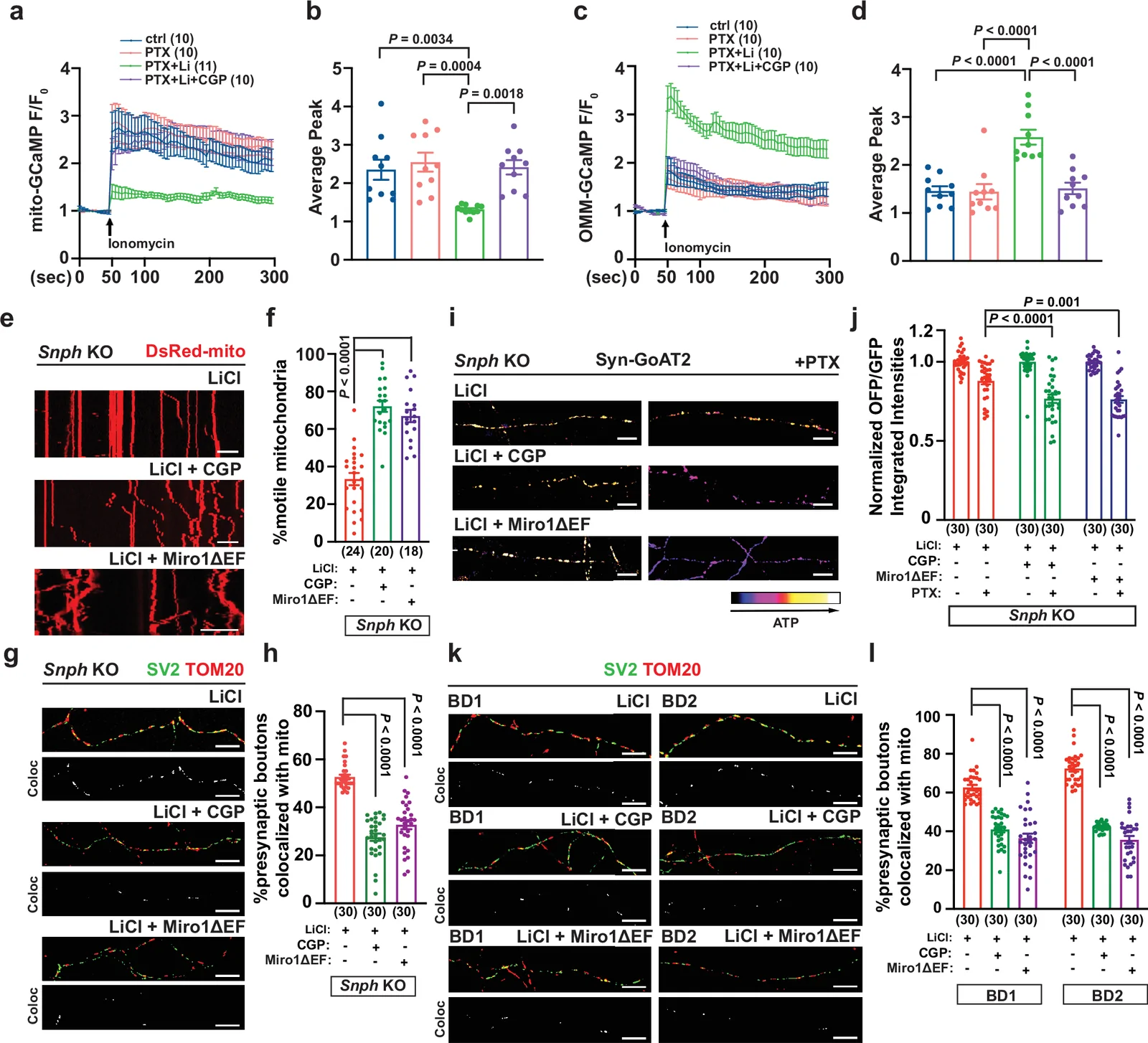
LiCl restores synaptoenergetics through the Na^+^/Ca^2+^/Li^+^ exchanger (NCLX). **a**–**d** LiCl enhances mitochondrial Ca^2+^ efflux (*P* < 0.0001) and Ca^2+^ microdomains at the mitochondrial surface (*P* < 0.0001). Neurons expressing a mitochondrial matrix-targeted calcium indicator (Mito-GCaMP5G) (**a**, **b**) or an outer mitochondrial membrane-targeted calcium indicator (OMM-GCaMP6f) (**c**, **d**) were treated with LiCl (100 µM) for 4 days. Prior to live imaging, neurons were preloaded for 4 h with PTX (100 µM) or the NCLX inhibitor CGP37157 (2 µM). Time-lapse imaging was performed following ionomycin (2 µM) addition. Normalized F/F_0_ traces illustrate calcium dynamics in mitochondrial matrix ([Ca^2+^]_matrix_; **a**) and surface ([Ca^2+^]_OMM_; **c**). **e**, **f** LiCl-induced arrest of axonal mitochondrial transport in *Snph* KO neurons is abolished by NCLX inhibition (*P* < 0.0001) or expression of the Ca^2+^-insensitive Miro1ΔEF mutant (*P* < 0.0001). Neurons were treated with DMSO or LiCl (100 µM) for 4 days (DIV10-14) and incubated with either DMSO or the NCLX inhibitor CGP37157 (2 µM) for 4 h prior to imaging at DIV14. **g**, **h** LiCl-mediated restoration of presynaptic mitochondrial retention in *Snph* KO neurons is prevented by NCLX inhibition (*P* < 0.0001) or expression of Miro1ΔEF (*P* < 0.0001). Colocalized SV2 and TOM20 signals are highlighted in binary images, indicate presynaptic mitochondria. **i**, **j** LiCl-mediated rescue of presynaptic ATP deficits in *Snph* KO neurons is abolished by NCLX inhibition (*P* < 0.0001) or expression of Miro1ΔEF (*P* < 0.001). The pseudocolor bar scale represents fluorescence intensity in arbitrary units (AU), with blue indicating low ATP and yellow indicating high ATP. **k**, **l** LiCl-enhanced presynaptic mitochondria retention in hiPSC-derived neurons from donors with bipolar disorder is abolished by NCLX inhibition (*P* < 0.0001) or expression of Miro1ΔEF (*P* < 0.0001). Representative images (**k**) and quantification (**l**). The colocalized pixels, highlighted in the white-black images, indicate presynaptic mitochondria. Data are presented as mean ± s.e.m. from the number of neurons indicated in parentheses across three independent experiments and were analyzed by one-way ANOVA with Tukey’s multiple-comparison test (**b**, **d**, **f**, **h**, **j**, **l**). Scale bars: 10 µm. Source data are provided as a Source Data file.

Mitochondrial Ca^2+^ transients are determined by the balance between rapid Ca^2+^ influx through the mitochondrial calcium uniporter (MCU)^76^ and slower Ca^2+^ efflux through the NCLX^77–81^. We therefore examined whether lithium regulates mitochondrial retention through NCLX-dependent Ca^2+^ efflux. Using the mitochondrial matrix-targeted Ca^2+^ indicator Mito-GCaMP5G, we monitored mitochondrial matrix Ca^2+^ ([Ca^2+^]_matrix_) following ionomycin stimulation. Resting mitochondrial Ca^2+^ levels did not differ between untreated and PTX-treated *Snph* KO neurons (*P* = 0.8979). PTX-induced neuronal activity alone did not significantly alter [Ca^2+^]_m_. In contrast, LiCl together with PTX significantly reduced [Ca^2+^]_matrix_ (*P* < 0.0001) and this effect was abolished by the NCLX inhibitor CGP37157 (2 μM) (*P* < 0.0001; Fig. 9a, b), consistent with increased NCLX-dependent mitochondrial Ca^2+^ efflux during sustained neuronal activity. We next monitored Ca^2+^ at the mitochondrial surface using the outer mitochondrial membrane-targeted Ca^2+^ indicator OMM-GCaMP6f. LiCl significantly increased mitochondrial surface Ca^2+^ signals during sustained neuronal activity (*P* < 0.0001), and this effect was also abolished by CGP37157 (*P* < 0.0001; Fig. 9c, d), indicating that lithium elevates mitochondrial surface Ca^2+^ microdomains in an NCLX-dependent manner.

To determine whether lithium-induced presynaptic mitochondrial retention requires NCLX-Miro1 signaling, *Snph* KO neurons were treated with CGP37157 or transfected with a Ca^2+^-insensitive Miro1 mutant (Miro1ΔEF, E208K/E3328K)^82^. Both NCLX inhibition and Miro1ΔEF expression abolished the lithium-induced reduction in axonal mitochondrial motility (*P* < 0.0001; Fig. 9e, f) and prevented restoration of presynaptic mitochondrial retention (*P* < 0.0001; Fig. 9g, h). Likewise, the lithium-mediated improvement in presynaptic ATP maintenance was blocked by CGP37157 (*P* < 0.0001) or Miro1ΔEF expression (*P* = 0.001; Fig. 9i, j).

The requirement for NCLX-Miro1 signaling was also observed in hiPSC-derived neurons from donors with bipolar disorder. Both CGP37157 treatment and Miro1ΔEF expression reduced lithium-mediated restoration of presynaptic mitochondrial retention (*P* < 0.0001; Fig. 9k, l) and attenuated the improvement in presynaptic ATP maintenance (Supplementary Fig. 8a–d). Similar results were obtained in AKAP11-deficient mouse neurons (Supplementary Fig. 8e–h).

**Fig. 10:**
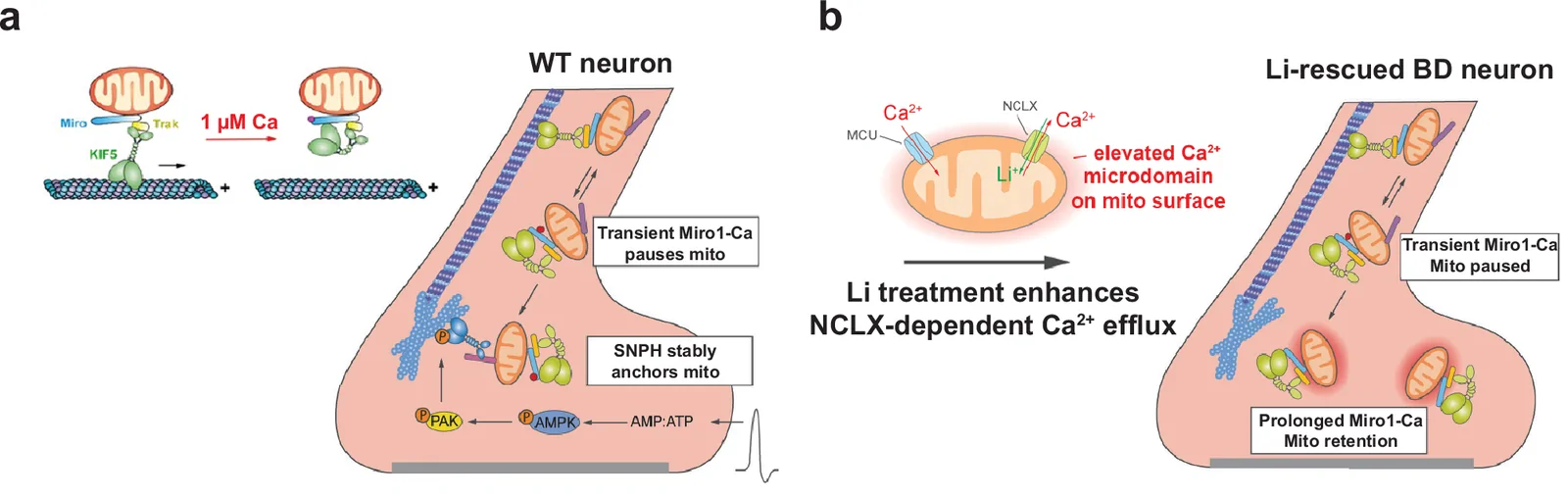
Proposed model: Lithium restores synaptoenergetics in neurons from donor with bipolar disorder and from *Snph* KO mice. **a** During synaptic activity, transient elevations in presynaptic Ca^2+^ pause motile mitochondria through Ca^2+^ binding to Miro1. This arrest is normally short-lived because Miro1 has relatively low Ca^2+^ affinity (~1 µM) and mitochondrial Ca^2+^ uptake rapidly dissipates local Ca^2+^ signals. Under physiological conditions, SNPH-mediated anchoring stabilizes these transient pauses by tethering mitochondrial to presynaptic actin, thereby maintaining local ATP supply. **b** In *Snph* KO neurons and hiPSC-derived neurons from donors with bipolar disorder, where SNPH expression is reduced, lithium restores presynaptic mitochondrial retention through an alternative mechanism. By promoting mitochondrial Ca^2+^ efflux via NCLX, lithium prolongs Ca^2+^ microdomains at the mitochondrial surface, sustaining Miro1-Ca^2+^ interactions and extending mitochondrial retention at presynaptic terminals independent of SNPH-mediated anchoring.

Together, these findings indicate that lithium promotes presynaptic mitochondrial retention through an NCLX-dependent mechanism that requires Miro1 Ca^2+^ sensing. This mechanism is associated with improved presynaptic ATP maintenance in both mouse and human bipolar disorder-relevant neuronal models where SNPH-mediated anchoring is compromised (Fig. 10).

## Discussion

This study examined whether axonal mitochondrial hypermotility contributes to synaptic variability and mood-related phenotypes associated with bipolar disorder and whether restoring presynaptic mitochondrial retention is associated with the therapeutic actions of lithium. We found that increased mitochondrial motility and reduced presynaptic mitochondrial retention in both hiPSC-derived neurons from donors with bipolar disorder and AKAP11-deficient mouse neurons were associated with accelerated activity-induced presynaptic ATP depletion and increased variability in synaptic transmission. Reduced SNPH expression in hiPSC-derived neurons from donors with bipolar disorder, together with rescue by SNPH re-expression and complementary analyses in *Snph* knockout mice, supports a role for impaired mitochondrial anchoring in these synaptoenergetic defects. Rather than producing uniform defects  in synaptic transmission, these alterations were characterized by increased fluctuations in presynaptic ATP availability and synaptic responses during sustained neuronal activity. Chronic lithium treatment restored presynaptic mitochondrial retention, improved ATP maintenance and Ca^2+^ buffering, reduced synaptic variability, and reversed mania-like behavioral phenotypes in *Snph* knockout mice. Mechanistically, these effects were associated with NCLX-dependent mitochondrial Ca^2+^ efflux and Miro1-mediated mitochondrial arrest, enabling presynaptic mitochondrial retention despite the absence of SNPH-mediated anchoring. Together, these findings support a model in which impaired presynaptic mitochondrial retention contributes to synaptoenergetic deficits and synaptic variability in bipolar disorder-relevant neuronal models and suggest that restoration of presynaptic mitochondrial retention is associated with the cellular and behavioral effects of lithium.

### Mitochondrial hypermotility contributes to bipolar disorder-associated synaptic variability

Altered energy metabolism is a consistent feature of bipolar disorder^15,83^. Analyses of post-mortem brain tissue and patient-derived cellular models have reported changes in mitochondrial bioenergetics, mtDNA copy number, and mitochondrial abundance^22–24,26^, suggesting altered neuronal energy metabolism. Because mitochondria supply approximately 93% of neuronal ATP, widespread mitochondrial dysfunction would be expected to impair neuronal energy metabolism broadly, potentially leading to synaptic loss and neurodegeneration. In contrast, our findings support a model in which increased axonal mitochondrial motility selectively disrupts presynaptic energy homeostasis during sustained neuronal activity without detectable changes in overall mitochondrial bioenergetic capacity.

Synaptic vesicle exocytosis and recycling are among the most energy-demanding processes in neurons, with recycling of a single glutamatergic synaptic vesicle estimated to require up to ~11,000 ATP molecules^84^. Although motile mitochondria distribute metabolic support throughout axons, stable mitochondrial retention at presynaptic terminals provides a local source of ATP and Ca^2+^ buffering required to sustain neurotransmission. Our results indicate that increased axonal mitochondrial motility reduces stable presynaptic mitochondrial residency, leading to accelerated activity-induced ATP depletion despite preserved mitochondrial bioenergetic capacity. These changes are consistent with impaired presynaptic energy maintenance contributing to increased variability in synaptic strength and altered short-term synaptic plasticity. Similar cellular phenotypes were observed in both hiPSC-derived neurons from donors with bipolar disorder and AKAP11-deficient mouse neurons. In addition, *Snph* KO mice, which exhibit increased axonal mitochondrial motility, displayed mania-like behavioral phenotypes, whereas reduced SNPH expression in hiPSC-derived neurons from donors with bipolar disorder and human post-mortem brain samples is consistent with impaired mitochondrial anchoring contributing to bipolar disorder-associated synaptoenergetic deficits. Together, these findings support a model in which increased axonal mitochondrial motility contributes to synaptoenergetic deficits, increased synaptic variability, and mania-like behavioral phenotypes.

Several observations further support a predominantly presynaptic mechanism. First, SNPH is an axon-specific mitochondrial anchoring protein that selectively regulates axonal mitochondrial anchoring. Second, synapse density and mean mEPSC amplitudes were unchanged (Supplementary Fig. 1; Fig. 2a, b), arguing against major alterations in postsynaptic structure or basal synaptic strength. Third, spontaneous excitatory synaptic transmission was preserved (Fig. 2d–f). Together, these findings suggest that the principal changes occur at presynaptic terminals. Consistent with this interpretation, live imaging demonstrated increased mitochondrial entry-exit transit events at individual presynaptic terminals in hiPSC-derived neurons from donors with bipolar disorder (Fig. 3). Our previous study showed that transient mitochondrial residency at presynaptic terminals increases fluctuations in presynaptic ATP availability and synaptic vesicle cycling, whereas restoring mitochondrial anchoring by SNPH re-expression stabilizes synaptic transmission^9^. Together, these findings support a model in which increased axonal mitochondrial motility reduces stable presynaptic mitochondrial retention, contributing to greater variability in synaptic transmission through fluctuations in local ATP availability and Ca^2+^ buffering.

### Bipolar disorder-relevant neurons exhibit a distinct form of presynaptic variability

HiPSC-derived neurons from donors with bipolar disorder exhibit increased synaptic variability associated with enhanced axonal mitochondrial motility. This pattern differs from that observed in many neurodegenerative disorders, which are typically characterized by progressive mitochondrial dysfunction, synaptic loss, or neuronal degeneration. Under a model of persistent mitochondrial depletion at presynaptic terminals, reduced mitochondrial Ca^2+^ buffering would be expected to increase presynaptic Ca^2+^ levels and enhance release probability. In contrast, our live-imaging analyses (Fig. 3) support a dynamic model in which increased axonal mitochondrial motility destabilizes local ATP availability and Ca^2+^ buffering during sustained neuronal activity. This instability is reflected by increased pulse-to-pulse variability in synaptic strength and enhanced sweep-to-sweep variability in paired-pulse ratio (PPR), rather than a persistent one-way increase or decrease in synaptic strength, leaving mean measures of synaptic transmission largely unchanged.

One implication of this model is that individual presynaptic terminals repeatedly transition between states with and without local mitochondrial support. When mitochondria are transiently retained at presynaptic terminals, local ATP supply and Ca^2+^ buffering support stable synaptic vesicle release and recycling. Conversely, when mitochondria move away from these sites, presynaptic ATP availability and Ca^2+^ buffering is transiently reduced. Repeated transitions between these states would be expected to increase trial-to-trial variability in synaptic transmission without substantially altering population- and time-averaged synaptic output. Consistent with this model, lithium restored presynaptic mitochondrial retention, reduced synaptic variability, and improved presynaptic ATP maintenance and Ca^2+^ buffering. Together, our live-imaging, electrophysiological, and metabolic analyses support a model in which increased axonal mitochondrial motility contributes to fluctuation-prone presynaptic function in bipolar disorder-relevant neurons.

### Synaptic fluctuations may constitute a chronic vulnerability to mood dysregulation

Our findings suggest that reduced SNPH expression in hiPSC-derived neurons from donors with bipolar disorder represents a persistent presynaptic alteration associated with increased axonal mitochondrial motility. Frequent mitochondrial entry-exit events at individual presynaptic terminals generate fluctuations in local ATP availability and Ca^2+^ buffering, resulting in increased variability in synaptic strength. Over prolonged periods, such variability could engage homeostatic and circuit-level adaptive mechanisms, potentially increasing susceptibility to mood dysregulation. Within this framework, rapid fluctuations in synaptic function may represent a chronic vulnerability, thereby biasing neural networks toward dysregulation, rather than directly determining the onset or timing of mood episodes. Instead, the emergence of manic or depressive episodes is likely governed by additional regulatory processes operating over longer timescales. Our findings identify impaired presynaptic mitochondrial retention and synaptoenergetic instability as cellular features associated with bipolar disorder and suggest that these alterations may contribute to mood-related phenotypes.

An integrated model of bipolar disorder proposes that alterations in neural network dynamics contribute to recurrent mood episodes^85^. Within these networks, presynaptic plasticity and stochasticity support information processing while accommodating fluctuating metabolic demands^86^. Stable mitochondrial retention at presynaptic terminals helps maintain local ATP supply and Ca^2+^ buffering, thereby supporting reliable synaptic transmission^9,87^. Reduced SNPH expression may disrupt this process by increasing axonal mitochondrial motility and reducing stable presynaptic mitochondrial retention. During sustained neuronal activity, these changes may promote local energetic fluctuations that influence synaptic stability. In both hiPSC-derived neurons from donors with bipolar disorder and *Snph* KO neurons, lithium restored presynaptic mitochondrial retention, improved ATP maintenance and Ca^2+^ buffering, and reduced synaptic variability through an SNPH-independent mechanism. These findings suggest that restoration of presynaptic mitochondrial retention contributes to the cellular actions of lithium and are consistent with its clinical efficacy in mitigating manic episodes^20^.

Previous studies have reported altered mitochondrial morphology and membrane potential in patient-derived dentate gyrus-like neurons^23^. Several factors may account for differences between those findings and ours. First, differences in patient cohorts, neuronal subtypes, and differentiation protocols are likely to influence neuronal maturation and metabolic state^88,89^. Second, our analyses were performed in more mature hiPSC-derived cortical neurons (DPI34), in which mitochondrial properties may differ from earlier developmental stages. Third, whereas previous studies primarily examined global mitochondrial properties, our work focused on activity-dependent mitochondrial dynamics in axons, including mitochondrial transport, presynaptic retention, and local ATP maintenance in live neurons. This perspective may be particularly relevant to bipolar disorder, which is characterized by episodic changes in mood and synaptic function. Finally, the heterogeneous clinical response to lithium suggests that multiple pathogenic mechanisms contribute to bipolar disorder. Our findings suggest that impaired presynaptic mitochondrial retention may represent one mechanism contributing to lithium-responsive forms of the disorder.

### *Snph* KO mice as a model for studying the manic phase of bipolar disorder

Early studies linking bipolar disorder to altered brain energy metabolism prompted investigations into mitochondrial dysfunction^24^. Although severe mitochondrial dysfunction can lead to synaptic failure and neurodegeneration^90^, bipolar disorder is primarily characterized by episodic changes in mood and synaptic function while neuronal viability is largely preserved. Our findings support a model in which impaired presynaptic mitochondrial retention, rather than global mitochondrial dysfunction, contributes to synaptoenergetic deficits in bipolar disorder. *Snph* KO mice provide a useful model for investigating these processes because they exhibit impaired presynaptic mitochondrial retention, activity-induced ATP depletion, increased synaptic variability, and mania-like behavioral phenotypes despite preserved overall mitochondrial bioenergetic capacity.

Previous work showed that SNPH recruits axonal mitochondria to presynaptic terminals during activity-induced energy stress through the AMPK–PAK–Myo6-SNPH signaling pathway^10^. Altered SNPH expression has also been reported in several neuropsychiatric disorders, including major depressive disorder (MDD)^91,92^. Transcriptomic studies have identified reduced *Snph* mRNA expression in the subgenual anterior cingulate cortex of individuals with MDD^93^, whereas another study reported increased *Snph* expression in subcortical tissue from individuals with major depression who died by suicide^94^. In addition, SNPH and Myo6 levels were reduced in neuron-derived extracellular vesicles from patients with MDD and increased following selective serotonin reuptake inhibitor treatment^95^. SNPH has also been implicated in schizophrenia through its interaction with the schizophrenia-associated protein DISC1^96,97^. In the present study, SNPH protein levels were reduced by approximately 50% in hiPSC-derived neurons from donors with bipolar disorder, consistent with recent proteomic analyses reporting reduced SNPH abundance in synaptosome-enriched fractions from post-mortem brains from donors with bipolar disorder and in *Akap11* KO mice^60^. Together, these observations suggest that altered SNPH expression may contribute to disrupted presynaptic mitochondrial retention across multiple neuropsychiatric disorders.

Progress in understanding bipolar disorder has been limited by the availability of disease models that recapitulate both cellular and behavioral phenotypes. In this study, hiPSC-derived neurons from donors with bipolar disorder and *Snph* KO mice exhibited similar synaptoenergetic deficits, including increased axonal mitochondrial motility, reduced presynaptic mitochondrial retention, activity-induced ATP depletion, and increased synaptic variability. *Snph* KO mice also displayed behavioral alterations consistent with the manic phase of bipolar disorder^46^, including hyperlocomotion, increased exploratory behavior, reduced anxiety- and depression-like behaviors, and impaired sensorimotor gating. Importantly, these behavioral phenotypes were reversed by chronic lithium treatment, supporting the predictive validity of the model. Although SNPH has not been identified as a bipolar disorder risk gene, the reduced SNPH expression and impaired presynaptic mitochondrial retention observed in patient-derived neurons is consistent with synaptoenergetic deficits associated with bipolar disorder.

Animal models of bipolar disorder are commonly evaluated based on three criteria: construct, face, and predictive validity^98^. *Snph* KO mice satisfy many of these criteria. First, they exhibit construct validity by recapitulating cellular phenotypes observed in patient-derived neurons, including increased axonal mitochondrial motility, reduced presynaptic mitochondrial retention, and activity-induced synaptoenergetic deficits. Second, they display face validity by exhibiting mania-like behavioral phenotypes. Third, they demonstrate predictive validity, as these behavioral abnormalities are reversed by lithium treatment. Together, these characteristics support the use of *Snph* KO mice as a complementary model for investigating the cellular and bioenergetic mechanisms associated with the manic phase of bipolar disorder.

### Restoring presynaptic mitochondrial support as a cellular action of lithium

Lithium is a first-line mood-stabilizer for the long-term management of bipolar disorder^99^. However, despite its clinical efficacy, the mechanisms underlying its anti-manic effects remain incompletely understood. Our findings indicate that lithium restores presynaptic mitochondrial retention in both human and mouse bipolar disorder-relevant neuronal models, improving presynaptic ATP maintenance and Ca^2+^ buffering during sustained neuronal activity. These cellular changes are associated with reduced synaptic variability and support a role for presynaptic mitochondrial retention in the actions of lithium. Previous studies have shown that lithium modulates multiple cellular pathways, including Na^+^/K^+^ channel activity, neuroinflammation, or GSK-3, CREB, and AKT signaling^100–104^. In addition, lithium has been reported to regulate synaptic vesicle recycling at glutamatergic synapses^105^. Our findings provide a mechanistic framework: by stabilizing presynaptic mitochondrial retention, lithium supports the high energetic demands of sustained synaptic transmission. By reducing synaptic variability within neural circuits, this mechanism may contribute to lithium’s mood-stabilizing effects.

Synaptic activity induces presynaptic Ca^2+^ elevations that transiently arrest axonal mitochondria through Ca^2+^ binding to the motor adaptor Miro1^73,74,106,107^. Upon Ca^2+^ binding, Miro disrupts the interaction between mitochondria, the molecular motor KIF5, and microtubules, leading to a transient pause in mitochondrial movement. However, Miro1–Ca^2+^ signaling alone is insufficient to stably retain mitochondria at presynaptic terminals. Brief synaptic Ca^2+^ influx induces only transient mitochondrial pausing^74^, likely reflecting rapid mitochondrial Ca^2+^ uptake and/or diffusion of Ca^2+^ away from the mitochondrial surface, which rapidly dissipates local Ca^2+^ signals. Consistent with this idea, intense synaptic activity fails to arrest axonal mitochondria in *Snph* KO hippocampal neurons^75^, highlighting the critical role of SNPH in stabilizing paused mitochondria at presynaptic sites. Together, these findings support a model, in which transient Miro1-mediated pausing is coordinated with SNPH-dependent anchoring to achieve stable mitochondrial retention at presynaptic terminals. An optogenetic study further supports this framework by identifying two stationary mitochondrial populations in axons—movable and immovable—corresponding to transiently paused versus stably anchored mitochondria, respecrively^108^. When SNPH-mediated anchoring is compromised, as observed in *Snph* KO mice and hiPSC-derived neurons from donors with bipolar disorder, an alternative mechanism may require for sustaining Miro-Ca^2+^ signaling. Our findings indicate that lithium enhances mitochondrial Ca^2+^ efflux through NCLX, thereby maintaining Ca^2+^ microdomains at the mitochondrial surface. This prolonged peri-mitochondrial Ca^2+^ signaling is consistent with sustained Miro1 activation and extended mitochondrial pausing in the absence of SNPH, promoting presynaptic mitochondrial retention.

Bipolar disorder is genetically and mechanistically heterogeneous, and activity-induced synaptoenergetic deficits are unlikely to account for all disease-associated phenotypes. Instead, our findings suggest that impaired presynaptic mitochondrial support represents one contributor to bipolar disorder pathophysiology. In *Snph* knockout mice, chronic lithium treatment improved mania-like behavioral phenotypes and restored presynaptic mitochondrial retention, whereas valproic acid did not affect these cellular or behavioral deficits. These findings are consistent with lithium and VPA acting through distinct cellular mechanisms. VPA is thought to exert its therapeutic effects primarily through modulation of GABAergic signaling, voltage-gated ion channels, and histone deacetylases^67,68^. The hiPSC-derived neuronal lines used in this study were generated from lithium-responsive donors, which may contribute to the efficacy of lithium observed in these models. Together, these findings illustrate how mechanistically defined cellular and animal models can help dissect treatment mechanisms and may facilitate the development of more targeted therapeutic strategies for bipolar disorder.

## Methods

### *Snph* mouse line

All animal experiments were performed in accordance with the National Institutes of Health (NIH) Guide for the Care and Use of Laboratory Animal. Animal Study Protocol (#1303) was approved by the Animal Care and Use Committee (ACUC) of the National Institute of Neurological Disorders and Stroke (NINDS), the National Institute on Deafness and other Communication Disorders (NIDCD), and National Center for Complementary and Integrative Health (NCCIH), NIH. *Snph*^−/−^ and *Snph*^+/−^ transgenic mouse lines were generated by targeted gene replacement in embryonic stem cells and maintained on a C57BL/6 J background, as previously described^12^. For all cell biology, electrophysiology, and behavioral experiments, *Snph*^+/−^ mice were intercrossed to generate *snph*^+/+^, *snph*^+/−^, and *snph*^−/−^ littermates for direct comparison within the same genetic background. To maintain a consistent genetic background, the *snph*^+/−^ colony was backcrossed to C57BL/6 J mice (Jackson Laboratory) every 3–5 generations, and this breeding strategy was maintained for more than 30 generations. Mice were group-housed (3–5 animals per cage) under standard conditions (12-h light/12-h dark cycle, lights on at 06:00; ambient temperature 70 °F; relative humidity 45%).

### Generation of hiPSC-derived cortical neurons from healthy donors and donors with bipolar disorder

The following cell lines were obtained from the NIGMS Human Genetic Cell Repository at the Coriell Institute for Medical Research: GM05915, GM05992, GM05224, GM05215, and GM07219. Collection of the data and biological specimens for inclusion in the repository was reviewed and approved by the local Institutional Review Boards (IRBs) of the respective collecting institutions under Office for Human Research Protections (OHRP)-approved Assurances. All human cells used in this study were collected from donors who provided written informed consent prior to sample collection. Primary fibroblasts were cultured in Advanced DMEM (Thermo Fisher Scientific) supplemented with 20% fetal bovine serum (FBS; HyClone). Human iPSC lines were generated using the StemRNA 3rd Gen Reprogramming Kit (ReproCELL). Fibroblasts were transfected with reprogramming RNAs encoding OCT4, SOX2, KLF4, c-MYC, NANOG, and LIN28 using Lipofectamine RNAiMAX (Thermo Fisher Scientific) for three consecutive days. Individual iPSC colonies were manually isolated 14 days after transfection and validated by live TRA-1-60 immunostaining. Stable human iPSC lines expressing doxycycline-inducible transcription factor NGN2 were generated using the PiggyBac transposon system, following the methods as described^47,48^. Briefly, human iPSCs were co-transfected with PB-TO-hNGN2 and EF1α-transposase plasmids using Lipofectamine Stem (Thermo Fisher Scientific) and selected with puromycin (Sigma-Aldrich) for 10–14 days. Human iPSCs were maintained in mTeSR Plus medium (STEMCELL Technologies) and passaged with ReLeSR (STEMCELL Technologies) upon reaching approximately 80% confluency.

### DNA constructs

AKAP11 shRNA glycerol stocks (TRCN0000241708, TRCN0000241709, and TRCN0000241710) were purchased from Sigma-Aldrich. Syn-GECO, Syn-GoATeam2, mito-GoATeam2, HA-SNPH, and tdTomato-synaptophysin were generated previously^10,51,109^. The following plasmids were obtained from the indicated investigators: mito-GCaMP5G (F. Polleux, Columbia University; Addgene plasmid #105009), OMM-GCaMP6f (T. Ryan, Cornell University; Addgene plasmid #127874), DsRed-mito (R. Youle, NINDS, NIH), Miro1ΔEF (E208K/E328K) (P. Aspenström, Uppsala University), PB-TO-hNGN2 (M. Ward, NINDS, NIH; Addgene plasmid #172115), EF1α-Transposase (M. Ward, NINDS, NIH), EGFP-Mito (D. Sabatini, Whitehead Institute for Biomedical Research), and the lentiviral packaging plasmids psPAX2 and pMD2.G (D. Trono, École Polytechnique Fédérale de Lausanne). The AAV5 vector pAAV-SYN1-HA-hM3D(Gq) (pOTTC1596) was obtained from Addgene^110^.

### Mouse cortical neuron cultures

Primary cortical neurons were prepared from embryonic day 18 (E18) wild-type and *Snph*^−/−^ mouse embryos of either sex. Cortices were dissociated with papain (Worthington Biochemical Corporation), and neurons were plated onto 12-mm poly-L-ornithine-coated glass coverslips (Deckgläser) in 24-well plates. Neurons were maintained in Neurobasal medium supplemented with 2% B-27, 0.5 mM GlutaMAX, 55 μM 2-mercaptoethanol (Thermo Fisher Scientific), 10% fetal bovine serum (HyClone), and 0.25 μg ml⁻¹ insulin (Sigma-Aldrich). After 24 h, half of the plating medium was replaced with feeding medium consisting of Neurobasal medium supplemented with 2% B-27, 0.5 mM GlutaMAX, and 5 μM 5-fluoro-2′-deoxyuridine to inhibit glial proliferation. Half of the culture medium was replaced every 3 days. Neurons were infected with lentivirus at DIV5 or transfected with the indicated constructs by calcium phosphate precipitation at DIV7–9. Imaging experiments were performed at DIV14 using a Zeiss LSM 880 Airyscan confocal microscope.

### Differentiation and maturation of human cortical neurons

Human iPSCs were dissociated with Accutase (STEMCELL Technologies) and plated on day 0 post-induction (DPI0) in induction medium consisting of Knockout DMEM/F-12 (Thermo Fisher Scientific), 1% N-2 Supplement, 2 mM GlutaMAX, 1× non-essential amino acids (NEAA), 2 μg ml⁻¹ laminin (Sigma-Aldrich), 2 μg ml⁻¹ doxycycline (Sigma-Aldrich), 10 ng ml⁻¹ each of brain-derived neurotrophic factor (BDNF), glial cell line-derived neurotrophic factor (GDNF), and neurotrophin-3 (NT-3) (PeproTech), and 10 μM Y-27632 (Tocris). The induction medium was replaced daily through DPI4. At DPI4, cells were dissociated with Accutase and replated onto poly-L-ornithine- and laminin-coated 12-mm glass coverslips in maturation medium consisting of BrainPhys medium (STEMCELL Technologies), 2% B-27 Plus Supplement, 10 ng ml⁻¹ each of BDNF, GDNF, and NT-3, 200 μM dibutyryl-cAMP, 200 nM ascorbic acid, 2 μg ml⁻¹ laminin, 2 μg ml⁻¹ doxycycline, 0.5 mM GlutaMAX, 10 mM glucose (Sigma-Aldrich), and 55 μM β-mercaptoethanol. At DPI10, cultures were switched to growth medium containing BrainPhys medium, 2% B-27 Plus Supplement, 1% N-2 Supplement-A, 5% fetal bovine serum (HyClone), 200 μM dibutyryl-cAMP, 200 nM ascorbic acid, 2 μg ml⁻¹ laminin, 2 μg ml⁻¹ doxycycline, 0.5 mM GlutaMAX, 55 μM β-mercaptoethanol, 10 mM glucose (Sigma-Aldrich), and 1 μM 5-fluoro-2′-deoxyuridine (Sigma-Aldrich). Half of the culture medium was replaced weekly.

For electrophysiological recordings, differentiated human neurons were plated onto primary mouse glial feeder layers prepared from E18 mouse cortices and maintained in MEM (Thermo Fisher Scientific) supplemented with fetal bovine serum (HyClone), G-5 Supplement, GlutaMAX, and NEAA. Glial cells were replated onto poly-L-ornithine- and Matrigel-coated coverslips at DIV7 and used as feeder layers for human neurons at DPI10. Electrophysiological recordings were performed at DPI30–38. For imaging experiments, human neurons were infected with lentivirus at DPI16 or transfected with the indicated constructs between DPI18 and DPI20 using calcium phosphate precipitation. Live-cell imaging was performed between DPI21-DPI24 or DPI36-DPI40 using a Zeiss LSM 880 Airyscan confocal microscope.

### Immunofluorescence

Mouse and human neurons were fixed in 4% paraformaldehyde (Electron Microscopy Sciences) containing 4% sucrose (Sigma-Aldrich) for 20 min at room temperature (RT), washed three times with PBS, permeabilized with 0.15% Triton X-100 for 15 min, and blocked for 1 h in PBS containing 4% bovine serum albumin (BSA) and 3% normal goat serum (Sigma-Aldrich). Cells were incubated overnight at 4 °C with primary antibodies diluted in blocking buffer. The following primary antibodies were used: rabbit anti-TOM20 (1:100; Santa Cruz Biotechnology), mouse anti-SV2 (1:2000; Developmental Studies Hybridoma Bank), rabbit anti-βIII-tubulin (1:5000; BioLegend), mouse anti-c-Fos (1:400; Santa Cruz Biotechnology), rabbit anti-HA (1:200; Cell Signaling Technology), and rabbit anti-PSD95 (1:100; Thermo Fisher Scientific). After washing three times with PBS, samples were incubated for 60 min at RT with Alexa Fluor 488- or Alexa Fluor 546-conjugated secondary antibodies (Thermo Fisher Scientific), washed with PBS, mounted in Fluoro-Gel (Electron Microscopy Sciences), and imaged.

### Live-cell imaging and image analysis

Transfected neurons were transferred to pre-warmed Hibernate E Low Fluorescence medium (BrainBits) supplemented with 2% B-27 and 0.5 mM GlutaMAX and maintained at 37 °C using an air-stream incubator. Live-cell imaging was performed using a Zeiss LSM 880 Airyscan confocal microscope equipped with a 40×/1.3 NA oil-immersion objective. Images were acquired at 1024 × 1024-pixel resolution and analyzed using ZEN 2.1 SP3 (Zeiss), FIJI, and ImageJ (NIH). For axonal mitochondrial transport, time-lapse images were acquired every 10 s for 10 min, and mitochondrial motility was analyzed using kymographs generated in ImageJ. Presynaptic and mitochondrial ATP levels were measured in neurons expressing Syn-GoATeam2 or mito-GoATeam2 by collecting emission signals at 505–550 nm and >545 nm, respectively as previously described^10^. Ratiometric images were generated using FIJI. For presynaptic mitochondrial transition analysis, dual-color time-lapse images were acquired every 5 s for 15 min. Kymographs were generated in ImageJ to analyze the axonal mitochondrial motility at individual presynaptic terminals. Ratiometric images were generated using FIJI. For presynaptic Ca^2+^ imaging, DIV13–14 cortical neurons expressing Syn-GECO were transferred to an RC-21BRFS stimulation chamber (Warner Instruments) containing modified Tyrode’s solution. Electrical field stimulation (100 Hz, 10 s) was delivered through platinum electrodes, and images were acquired every second for 40 s. For mitochondrial matrix and mitochondrial surface Ca^2+^ imaging, DIV9 cortical neurons expressing mito-GCaMP5G or OMM-GCaMP6f were imaged in modified Tyrode’s solution every 5 s for 60 frames. Ionomycin (2 μM; Sigma-Aldrich) was added immediately after acquisition of the tenth frame. Mitochondrial membrane potential (Δψm) was assessed using two independent approaches. Neurons were loaded with MitoTracker Orange CMTMRos (50 nM; Thermo Fisher Scientific) for 30 min, fixed, immunostained for TOM20, and CMTMRos fluorescence was quantified within TOM20-positive mitochondria. Alternatively, live neurons were co-loaded with TMRE (25 nM) and MitoTracker Green (20 nM; Thermo Fisher Scientific) for 20 min before imaging. Δψm was quantified as TMRE fluorescence normalized to MitoTracker Green using ImageJ.

### Seahorse extracellular flux analysis

Mitochondrial respiration was assessed by measuring the oxygen consumption rate (OCR) using a Seahorse XFe96 Extracellular Flux Analyzer and the Seahorse XF Cell Mito Stress Test Kit (Agilent, 103015-100) according to the manufacturer’s instructions. Human neurons were plated at 50,000 cells per well. Before the assay, cultures were washed with pre-warmed artificial cerebrospinal fluid (ACSF; 120 mM NaCl, 3.5 mM KCl, 1.3 mM CaCl₂, 0.4 mM KH₂PO₄, 1 mM MgCl₂, 5 mM HEPES, 10 mM sodium pyruvate, and 10 mM glucose, pH 7.4) and equilibrated for 1 h at 37 °C in a CO₂-free incubator. OCR was measured under basal conditions and following sequential injections of oligomycin (1.0 μM), FCCP (1.5 μM), and rotenone/antimycin A (0.5 μM). Basal respiration, maximal respiration, spare respiratory capacity, and ATP-linked respiration were calculated using the Seahorse XF Cell Mito Stress Test Report Generator (Agilent). OCR values were normalized to cell number using a Cytation 5 Cell Imaging Multi-Mode Reader (Agilent).

### Immunoblotting

Cells were lysed in RIPA buffer (50 mM Tris-HCl, pH 7.4, 150 mM NaCl, 1% NP-40, 0.1% SDS, 0.5% sodium deoxycholate, and 2 mM EDTA) supplemented with cOmplete™ protease inhibitor cocktail (Roche). Equal amounts of protein were separated on 4–12% Bis-Tris NuPAGE gels (Thermo Fisher Scientific), transferred to PVDF membranes, and processed for immunoblotting. Primary antibodies were rabbit anti-SNPH (1:2000; Abcam), mouse anti-GAPDH (1:5000; Millipore), and rabbit anti-AKAP11 (1:1000; Novus Biologicals). HRP-conjugated secondary antibodies were mouse IgG (1:5000; GE Healthcare) and rabbit IgG (1:2500; GE Healthcare).

### Lentivirus production and neuronal infection

HEK293T cells were maintained in DMEM supplemented with 10% fetal bovine serum and 0.5 mM GlutaMAX. Lentiviral particles were generated by co-transfecting HEK293T cells with the transfer vector, psPAX2, and pMD2.G plasmids at a 4:3:1 ratio using Lipofectamine 2000 (Thermo Fisher Scientific). Culture medium was replaced the following day, and viral supernatants were collected 48–72 h after transfection, cleared by centrifugation (800 × *g*, 10 min), filtered through a 0.45-μm membrane, and concentrated by ultracentrifugation (90,000 × *g*, 90 min). Viral pellets were resuspended in PBS, aliquoted, stored at −80 °C, and used to infect cortical neurons at DIV4–7.

### Electrophysiology

Whole-cell patch-clamp recordings were performed on primary mouse cortical neurons (DIV14–19) and hiPSC-derived cortical neurons cultured on mouse glial feeder layers (DPI30–38). Coverslips were transferred to a recording chamber mounted on an upright microscope (BX50WI, Olympus) and continuously perfused with oxygenated artificial cerebrospinal fluid (ACSF). For mouse cortical neurons, ACSF contained (in mM): 125 NaCl, 25 NaHCO₃, 2.5 KCl, 1.25 NaH₂PO₄, 10 D-glucose, 2 MgCl₂, and 2 CaCl₂, equilibrated with 95% O₂/5% CO₂. Patch pipettes (4–8 MΩ; Sutter Instrument) were filled with an internal solution containing (in mM): 146.5 K-gluconate, 7.5 KCl, 9 NaCl, 1 MgCl₂, 10 HEPES, and 0.2 EGTA (pH 7.3, 295 mOsm). Dual whole-cell recordings were obtained from synaptically connected neuron pairs identified under current clamp by evoking action potentials with 400-pA current injections. Only neurons with resting membrane potentials of approximately −60 mV were included.

Pulse-to-pulse synaptic variability was assessed by eliciting presynaptic action potentials every 20 s (0.05 Hz) for 10 min using brief depolarizing pulses (−70 to +30 mV, 2 ms). The coefficient of variation (CV) was calculated as the standard deviation divided by the mean EPSC amplitude for each neuron. Short-term synaptic depression was examined using 20-Hz (20 action potentials, 1 s) or 50-Hz (10 action potentials, 200 ms) stimulation trains. Synaptic recovery was measured following a 2-s, 100-Hz high-frequency stimulation train, with single test stimuli delivered 1, 2, 5, and 10 s after stimulation. Recovery protocols were repeated three times at 120-s intervals. For lithium rescue experiments, neurons were treated with LiCl (100 μM) from DIV10 until recording.

For hiPSC-derived cortical neurons, the extracellular recording solution contained (in mM): 145 NaCl, 5 KCl, 2 MgCl₂, 3 CaCl₂, 10 HEPES, and 10 D-glucose (pH 7.4, 305 mOsm). Miniature and spontaneous EPSCs were recorded using Cs-based internal solution containing (in mM): 130 CsMeSO₃, 2.8 NaCl, 5 TEA-Cl, 20 HEPES, 0.4 EGTA, 2.5 MgATP, 0.25 Na₃GTP, and 5 QX-314 (pH 7.2–7.4, 285–295 mOsm). Tetrodotoxin (TTX, 1 μM) was included in the bath solution for mEPSC recordings. Evoked EPSCs were recorded using the K-gluconate-based internal solution described above. Synaptic responses were evoked with a bipolar stimulating electrode (World Precision Instruments) positioned approximately 200 μm from the recorded neuron. Stimulation protocols included 0.05-Hz stimulation for 10 min to assess pulse-to-pulse variability, paired-pulse stimulation with a 50-ms interstimulus interval, and 20-Hz stimulation trains (500 ms) to examine short-term synaptic depression. Signals were recorded using a MultiClamp 700B amplifier (Molecular Devices), filtered at 2 kHz, digitized at 20 kHz with a Digidata 1440 A interface (Molecular Devices), and acquired using pCLAMP 10.2 software. Cells exhibiting >20% changes in series resistance during recording were excluded from analysis.

### CNO-induced neuronal excitability

To assess the effects of clozapine-*N*-oxide (CNO; Tocris) on neuronal excitability, mouse cortical neurons were infected with AAV-hM3D(Gq) at DIV7 and recorded at DIV14. CNO (10 μM) was dissolved in oxygenated ACSF (95% O₂/5% CO₂) and perfused through the recording chamber during the experiment. For current-clamp recordings, patch pipettes (4–8 MΩ) were filled with an internal solution containing (in mM): 130 potassium gluconate, 6 NaCl, 20 HEPES, 0.2 EGTA, 1 MgCl₂, 2 MgATP, and 0.3 Na₃GTP (pH 7.2–7.4 with KOH; 285–295 mOsm). Spontaneous action potential firing was recorded in gap-free mode for 5 min before and after CNO application. Intrinsic neuronal excitability was assessed by injecting depolarizing current steps (500 ms, 50–300 pA in 50-pA increments) at 10-s intervals before and 10 min after CNO treatment.

### Mood behavioral analysis

To maintain a homogeneous experimental cohort during the initial behavioral characterization, only male mice (2–4 months old) from at least two independent cohorts were used for the behavioral experiments. Future studies including both sexes will be required to determine the generalizability of these findings. Mice were age-matched within each experiment. Behavioral testing was performed between 09:00 and 18:00 in sound-attenuated rooms at the Rodent Behavioral Core Facility (National Institute of Mental Health, NIH). Mice were acclimated to the testing room for 1 h before testing, and all apparatus were cleaned with 70% ethanol between animals. For lithium treatment experiments, 8-week-old WT and *Snph*^−/−^ mice received daily intraperitoneal injections of saline, lithium chloride (LiCl; 90 mg kg⁻¹), or valproic acid (VPA; 400 mg kg⁻¹) for 4 weeks. Behavioral testing was performed between 12 and 16 weeks of age with 1–2-day intervals between assays.

1. **Open-field test.** Locomotor activity was assessed in an automated open-field chamber (San Diego Instruments). The test was performed under dark conditions using infrared illumination (637 nm), allowing behavior to be recorded with an infrared camera. The center area of the chamber was defined as a 15 × 10 cm zone. Each mouse was placed in a corner of the chamber and allowed to explore freely for 60 min. Horizontal locomotor activity was quantified by X- and Y-axis photobeam breaks, and vertical activity (rearing) was quantified by Z-axis photobeam breaks. Tracking began with the first locomotor beam break, and an automated activity monitoring system continuously recorded horizontal locomotion and vertical rearing throughout the 60-min test.
2. **Elevated plus maze test.** The elevated plus maze consisted of two enclosed arms and two open arms arranged in a plus-shaped configuration. The maze was elevated 62.23 cm above the floor. Each mouse was placed in the center of the maze and allowed to explore freely for 10 min. An arm entry was scored only when at least 85% of the mouse’s body had entered the arm. Mouse behavior was monitored and analyzed using TopScan behavioral tracking software (CleverSys).
3. **Light-dark box test.** The light-dark box consisted of two compartments: a lit, open compartment and a dark, enclosed compartment, separated by a central entryway (4 cm W × 5 cm H). Each mouse was placed in the center of the light compartment and allowed to move freely between the two compartments for 15 min. The time spent in the light and dark compartments was recorded. Mouse behavior was monitored and analyzed using TopScan behavioral tracking software.
4. **Forced swim test.** Mice were individually placed in a Plexiglas cylinder (20 cm height × 10 cm diameter) filled with water (23 °C) to a depth of 7.5 cm and allowed to swim for 6 min. Total immobility time, the number of immobile episodes, and the latency to the first immobile episode were quantified using ANY-maze software (Stoelting).
5. **Prepulse inhibition test.** Acoustic startle response (ASR) and prepulse inhibition (PPI) were assessed using SR-LAB startle chambers (San Diego Instruments, San Diego, CA). Each chamber contained a clear, non-restrictive Plexiglas cylinder mounted on a platform within a ventilated enclosure with continuous 66-dB background white noise. The ASR session consisted of pulse-alone trials (80, 90, 100, 110, or 120 dB; 40 ms) and no-stimulus (NS) trials. The PPI session consisted of pulse-alone trials (120 dB, 40 ms), prepulse + pulse trials, and NS trials. In prepulse + pulse trials, a 20-ms prepulse (69, 73, or 81 dB) preceded the 120-dB, 40-ms startle pulse by 80 ms. The intertrial interval averaged 21 s (range, 12–30 s). The startle response was recorded for each trial, and percent prepulse inhibition (%PPI) was calculated as:

$$\%{{{\rm{PPI}}}}=100-[({{{\rm{prepulse}}}}+{{{\rm{pulse\; response}}}})/({{{\rm{pulse}}}}\mbox-{{{\rm{alone\; response}}}})\times 100].$$

### Measurement of lithium and VPA concentrations

Brain tissue and serum samples were collected from WT and *Snph* KO mice before and after drug administration. Total lithium concentrations were measured by inductively coupled plasma mass spectrometry (ICP-MS)^100^. Serum valproic acid (VPA) concentrations were determined by low-resolution electrospray ionization mass spectrometry (LRESIMS). LRESIMS analyses were performed using an Agilent 1260 Infinity II LC system coupled to an Agilent 6310 MSD. The HPLC column was obtained from Waters, and all HPLC-grade solvents were purchased from Sigma-Aldrich.

### Statistics and reproducibility

Quantification was performed without blinding. Statistical details, including the definition of *n* (number of biologically independent neurons, mice, or independent experiments), sample sizes, statistical tests, and *P* values, are provided in the corresponding figure legends. All experiments were independently repeated at least three times unless otherwise indicated. When appropriate, statistical analyses were performed using biologically independent replicates. Statistical analyses were performed using GraphPad Prism 9 (GraphPad Software). Comparisons between two groups were analyzed using two-sided unpaired Student’s *t*-tests or two-sided Mann–Whitney tests, as appropriate. Comparisons among three or more groups were analyzed using one-way ANOVA followed by Dunnett’s multiple-comparison test when comparing experimental groups with a single control or Tukey’s multiple-comparison test when comparing all groups. Grouped datasets were analyzed using two-way ANOVA followed by Holm–Šidák multiple-comparison tests, as indicated. Data are presented as mean ± s.e.m., and differences were considered statistically significant at *P* < 0.05.

### Reporting summary

Further information on research design is available in the Nature Portfolio Reporting Summary linked to this article.

## Supplementary information

Supplementary Information_Suppl Figures and Table

Reporting summary

Transparent Peer Review file

## Source data

source data

## Data Availability

All data supporting the findings of this study are provided in the Supplementary Information/Source Data file. Because of their very large file size, the original microscopy images and time-lapse live images, and videos recording behavioral phenotypes cannot be publicly deposited and are available from the corresponding author upon request. Any additional information required to reanalyze the data reported in this paper is available from the corresponding author upon request. Source data are provided with this paper.
