# Supplementary material for: Enhanced axonal mitochondrial motility and neural activity-induced energy deficits destabilize synaptic transmission in models of bipolar disorder
Source: Nat Commun. 2026 Aug 22;17:10159. doi: 10.1038/s41467-026-76722-x (PMC13601522; doi:10.1038/s41467-026-76722-x)
Supplement: Supplementary file 1 [file 41467_2026_76722_MOESM1_ESM.pdf]

Supplementary Information

Enhanced axonal mitochondrial motility and neural activity-induced energy deficits destabilize synaptic transmission in models of bipolar disorder

Sunan Li [1, #], Gui-Jing Xiong[1, #], Zezhi Li[1, #], Zu-Hang Sheng[1, *]

[1,] Synaptic Function Section, The Porter Neuroscience Research Center, National Institute of Neurological Disorders and Stroke (NINDS), National Institutes of Health, Room 2B-215, 35 Convent Drive, Bethesda, Maryland 20892-3706, USA.

[#] These authors contributed equally.

* Correspondence should be addressed to Z.-H. Sheng (shengz@ninds.nih.gov)

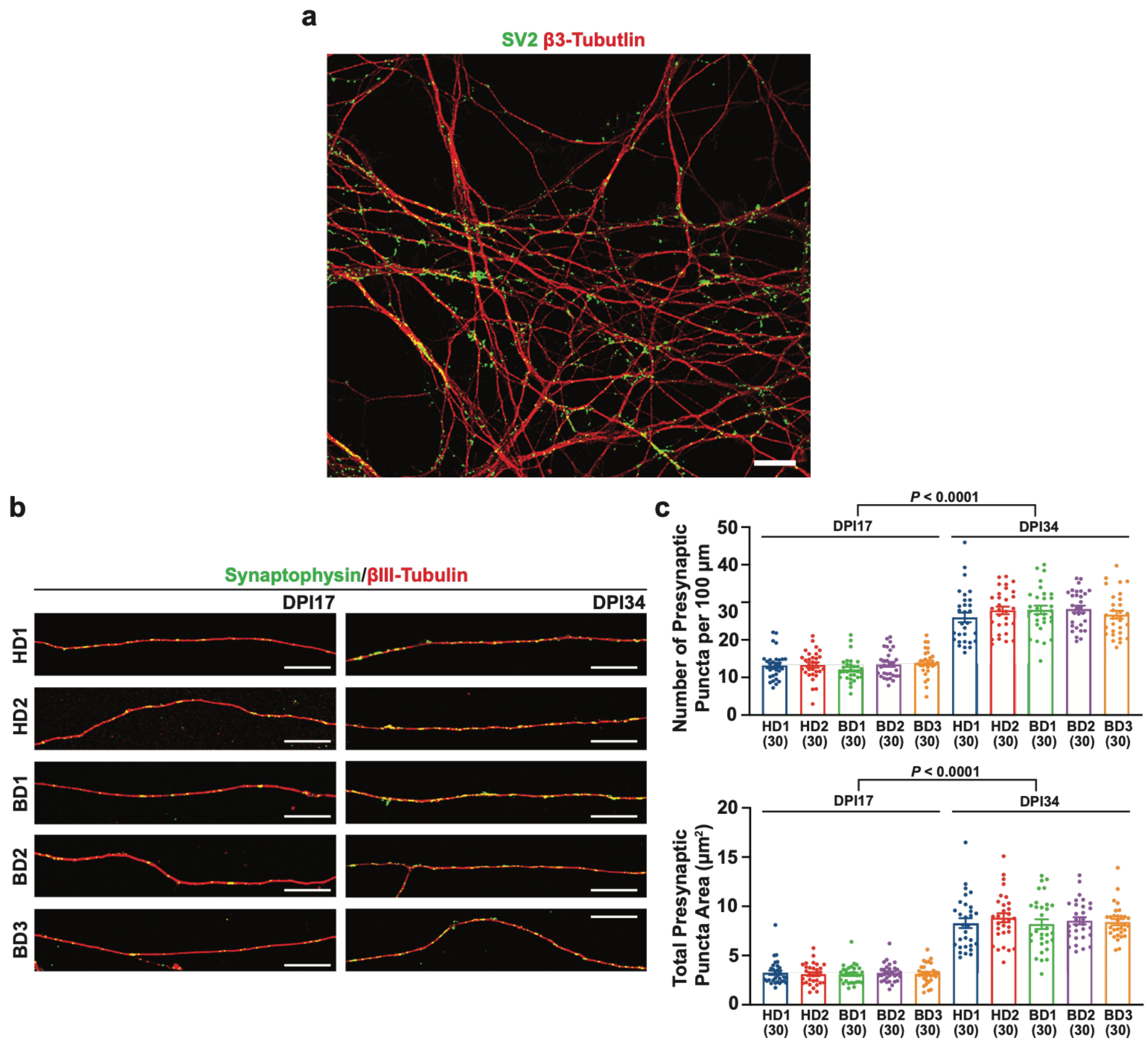
a
SV2 β3-Tubutlin
b
Synaptophysin/βIII-Tubulin
DPI17
DPI34
HD1
HD2
BD1
BD2
BD3
c
P < 0.0001
DPI17
DPI34
Number of Presynaptic Puncta per 100 µm
50
40
30
20
10
0
HD1 (30) HD2 (30) BD1 (30) BD2 (30) BD3 (30) HD1 (30) HD2 (30) BD1 (30) BD2 (30) BD3 (30)
P < 0.0001
DPI17
DPI34
Total Presynaptic Puncta Area (µm²)
20
15
10
5
0
HD1 (30) HD2 (30) BD1 (30) BD2 (30) BD3 (30) HD1 (30) HD2 (30) BD1 (30) BD2 (30) BD3 (30)

Supplementary Fig. 1. Synaptic development in hiPSC-derived cortical neurons from healthy donors and donors with bipolar disorder.
a, Representative image of NGN2-induced hiPSC-derived cortical neurons at DPI21 co-immunostained for the presynaptic marker SV2 and the neuronal marker β3-tubulin.
b, c, Synaptic development in cortical neurons derived from healthy donors (HD1 and HD2) and donors with bipolar disorder (BD1-3). Neurons were immunostained for synaptophysin and β3-tubulin at DPI17 and DPI34. Synapse density and synaptic area along axons increased significantly between DPI17 and DPI34 ($P < 0.0001$), indicating progressive synaptic maturation. No significant differences in synapse density were detected between healthy donor and bipolar disorder neurons at either time point. Data are presented as mean ± s.e.m. from the number of neurons indicated in parentheses across three independent experiments and were analyzed using one-way ANOVA. Scale bar, 10 μm. Source data are provided as a Source Data file.

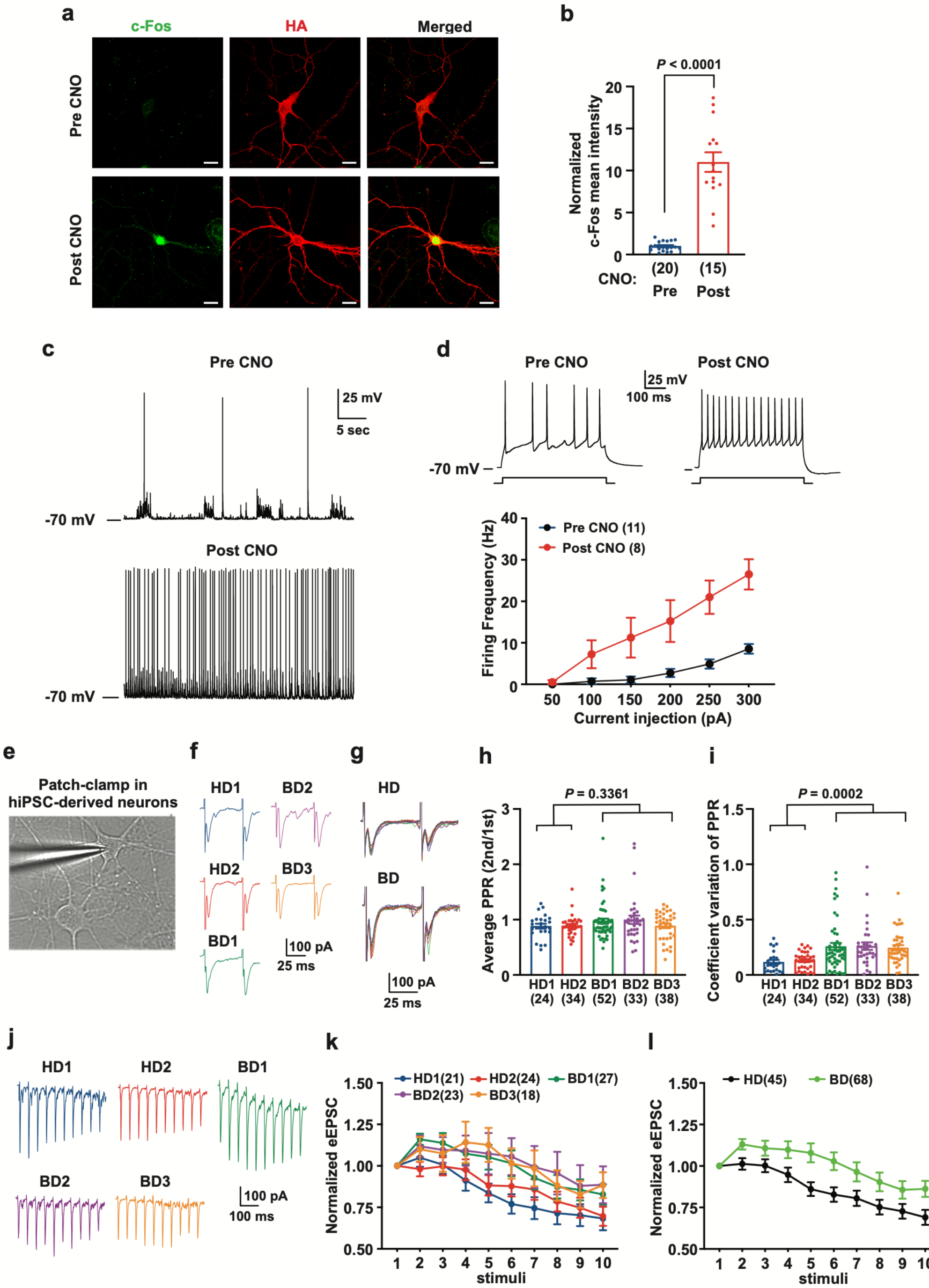
a
c-Fos
HA
Merged
Pre CNO
Post CNO
b
P < 0.0001
Normalized c-Fos mean intensity
CNO: (20) Pre (15) Post
c
Pre CNO
25 mV
5 sec
-70 mV
Post CNO
-70 mV
d
Pre CNO
Post CNO
25 mV
100 ms
-70 mV
Pre CNO (11)
Post CNO (8)
Firing Frequency (Hz)
Current injection (pA)
e
Patch-clamp in hiPSC-derived neurons
f
HD1
BD2
HD2
BD3
BD1
100 pA
25 ms
g
HD
BD
100 pA
25 ms
h
P = 0.3361
Average PPR (2nd/1st)
HD1 (24) HD2 (34) BD1 (52) BD2 (33) BD3 (38)
i
P = 0.0002
Coefficient variation of PPR
HD1 (24) HD2 (34) BD1 (52) BD2 (33) BD3 (38)
j
HD1
HD2
BD1
BD2
BD3
100 pA
100 ms
k
HD1(21) HD2(24) BD1(27) BD2(23) BD3(18)
Normalized eEPSC
stimuli
l
HD(45) BD(68)
Normalized eEPSC
stimuli

Supplementary Fig. 2. hiPSC-derived cortical neurons from donors with bipolar disorder exhibit increased sweep-to-sweep variability in paired-pulse ratio.

a, b, Chemogenetic activation increases neuronal activity. Representative images (**a**) and quantification (**b**) of c-Fos expression in hiPSC-derived cortical neurons expressing HA-hM3D(Gq) following CNO treatment (10 μM for 30 min) at DPI28. The mean intensity of c-Fos was normalized to neurons before CNO treatment.

c, d, Chemogenetic activation enhances excitability in mouse cortical neurons. Neurons were infected with AAV5-hSyn1-HA-hM3D(Gq) at DIV7 and analyzed at DIV14. Representative action potential traces (**c**) and firing frequency-current relationship (**d**) showing increased neuronal firing following CNO treatment (two-way ANOVA: $F_{1,17}$ = 14.40, P = 0.0014).

e–i, Increased sweep-to-sweep variability in paired-pulse ratio (PPR) in hiPSC-derived cortical neurons from donors with bipolar disorder. Recording configuration (**e**), stimulation paradigm (**f**), and paired-pulse evoked EPSC traces (5-10 sweeps per neuron) (**g**). Mean PPR did not differ between hiPSC-derived neurons from HD and BD donors ($F_{4, 176}$ = 1.147, P = 0.3361) (**f, h**). In contrast, neurons from BD donors exhibited significantly greater sweep-to-sweep variability in PPRs, reflected by an increased coefficient of variation ($F_{4, 176}$ = 5.830, P = 0.0002) (**g, i**).

j-l, Reduced short-term synaptic depression in neurons derived from donors with bipolar disorder under physiological extracellular Ca^{2+}. EPSCs were evoked by a 20-Hz, 500-ms stimulus train in 1.3 mM extracellular Ca^{2+} (**j**). Normalized eEPSC amplitudes were plotted against the first stimulus in the train. Neurons grouped from donors with bipolar disorder exhibited significantly slower short-term depression than healthy donor neurons (**l**; two-way ANOVA: group effect $F_{1, 111}$ = 5.639, P = 0.0193; stimulus x group interaction $F_{9, 999}$ = 2.947, P = 0.0018).

Data are presented as mean ± s.e.m. from the number of individual neurons indicated in parentheses across three independent experiments and were analyzed using the two-tailed Mann-Whitney test (**b**), one-way ANOVA (**h, i**), or two-way ANOVA with Tukey's post-hoc test (**d**, **k, l**). Scale bars: 20 μm. Source data are provided as a Source Data file.

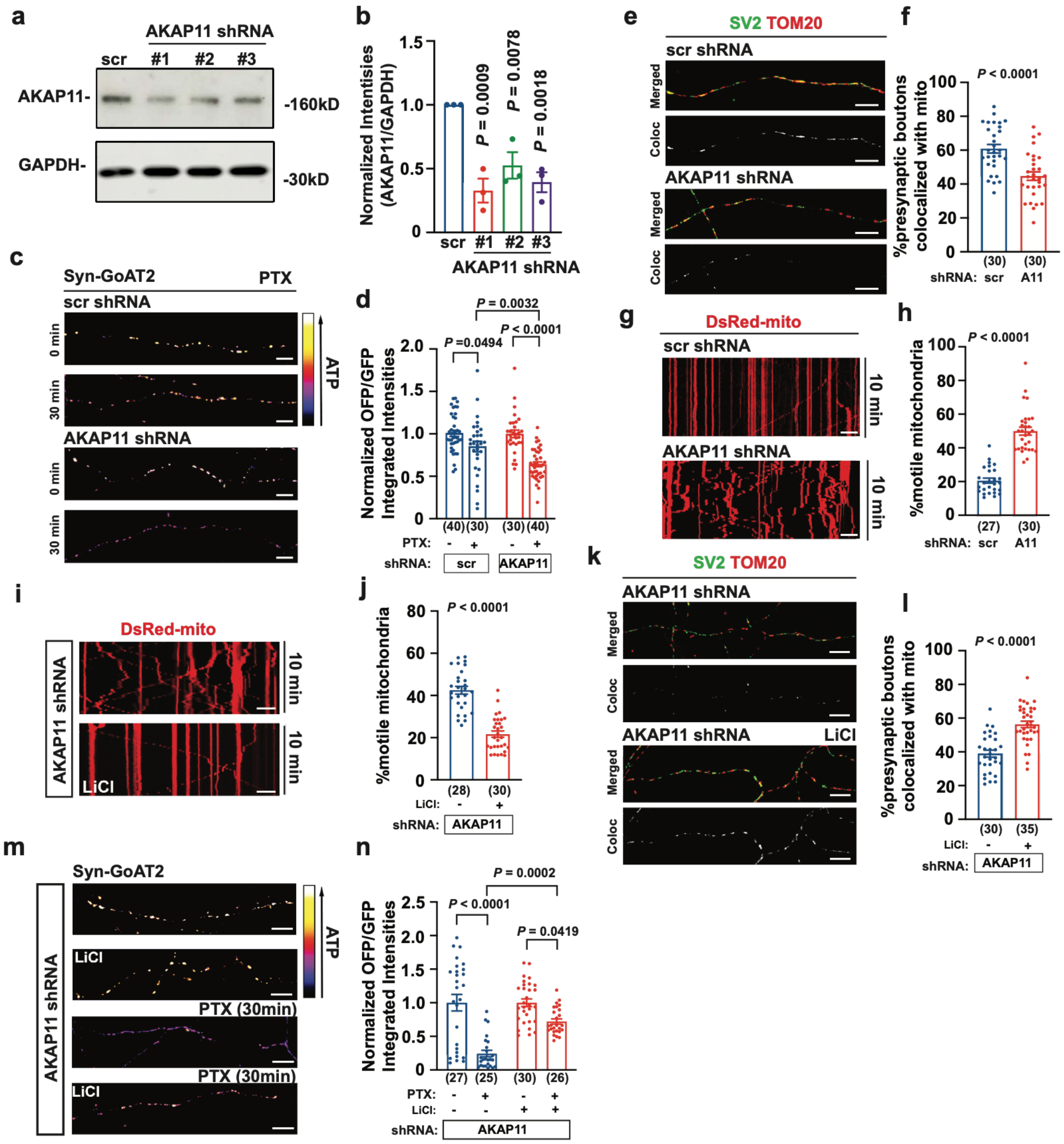
a
AKAP11 shRNA
scr #1 #2 #3
AKAP11-
-160kD
GAPDH-
-30kD
b
Normalized Intensities (AKAP11/GAPDH)
P = 0.0009
P = 0.0078
P = 0.0018
scr #1 #2 #3
AKAP11 shRNA
c
Syn-GoAT2
PTX
scr shRNA
0 min
30 min
AKAP11 shRNA
ATP
d
Normalized OFP/GFP Integrated Intensities
P = 0.0032
P < 0.0001
P =0.0494
(40) (30) (30) (40)
PTX:
shRNA: scr AKAP11
e
SV2 TOM20
scr shRNA
Merged
Coloc
AKAP11 shRNA
f
%presynaptic boutons colocalized with mito
P < 0.0001
(30) (30)
shRNA: scr A11
g
DsRed-mito
scr shRNA
AKAP11 shRNA
10 min
h
%motile mitochondria
P < 0.0001
(27) (30)
shRNA: scr A11
i
DsRed-mito
AKAP11 shRNA
LiCl
10 min
j
%motile mitochondria
P < 0.0001
(28) (30)
LiCl:
shRNA: AKAP11
k
SV2 TOM20
AKAP11 shRNA
Merged
Coloc
LiCl
l
%presynaptic boutons colocalized with mito
P < 0.0001
(30) (35)
LiCl:
shRNA: AKAP11
m
Syn-GoAT2
AKAP11 shRNA
LiCl
PTX (30min)
ATP
n
Normalized OFP/GFP Integrated Intensities
P = 0.0002
P < 0.0001
P = 0.0419
(27) (25) (30) (26)
PTX:
LiCl:
shRNA: AKAP11

Supplementary Fig. 3. AKAP11-deficient neurons exhibit synaptoenergetic deficits.
a, b, Validation of AKAP11 knockdown in mouse cortical neurons expressing three AKAP11 shRNAs. Immunoblots (**a**) and quantification of AKAP11 protein levels normalized to GAPDH and expressed relative to scramble (scr)-shRNA from three experiments (**b**). A pooled mixture of three AKAP11 shRNAs was used in subsequent experiments.
c, d, AKAP11-deficient neurons exhibit reduced presynaptic ATP levels during sustained activity. Neurons were co-transfected at DIV9 with Syn-GoATeam2 and scr-shRNA or AKAP11-shRNA, followed by imaging before and after PTX (100 μM, 30 min). Syn-GoATeam2 ATP heatmaps (**c**) and quantification of presynaptic ATP levels (**d**).
e, f, AKAP11-deficiency reduces presynaptic mitochondrial retention. Neurons expressing scr-shRNA (scr) or AKAP11-shRNA (A11) were immunostained for SV2 and TOM20. Colocalized pixels shown in white in the binary images indicate presynaptic mitochondria.
g, h, AKAP11-deficient neurons exhibit increased axonal mitochondrial motility. Representative kymographs (**g**) and quantification of motile axonal mitochondria (**h**).
i, j, Lithium reverses increased axonal mitochondrial motility in AKAP11-deficient neurons. Kymographs (**i**) and quantification of motile mitochondria (**j**). Neurons expressing AKAP11-shRNA were transfected with DsRed-Mito at DIV9 and treated with DMSO or LiCl (100 μM) for four days, followed by time-lapse imaging for 10 min at DIV14.
k, l, Lithium restores presynaptic mitochondrial retention in AKAP11-deficient neurons. Neurons expressing AKAP11-shRNA were treated with DMSO or LiCl (100 μM) for four days starting at DIV10, and co-immunostained for SV2 and TOM20 at DIV14.
m, n, Lithium mitigates activity-induced declines in presynaptic ATP levels in AKAP11-deficient neurons. Neurons expressing AKAP11-shRNA were transfected with Syn-GoATeam2 at DIV9 and treated with DMSO or LiCl (100 μM) for four days. Live imaging was performed at DIV14 before and after 30-min incubation with PTX (100 μM). The OFP/GFP ratio heatmaps reflects relative presynaptic ATP levels.
Data are presented as mean ± s.e.m. from the number of neurons indicated in parentheses across three independent experiments. Statistical analyses were performed using one-way ANOVA with Dunnett's (**b**) or Tukey's (**d**, **n**) tests, Student's *t*-test (**f**, **l**), or the Mann–Whitney test (**h**, **j**). Scale bars, 10 μm. Source data are provided as a Source Data file.

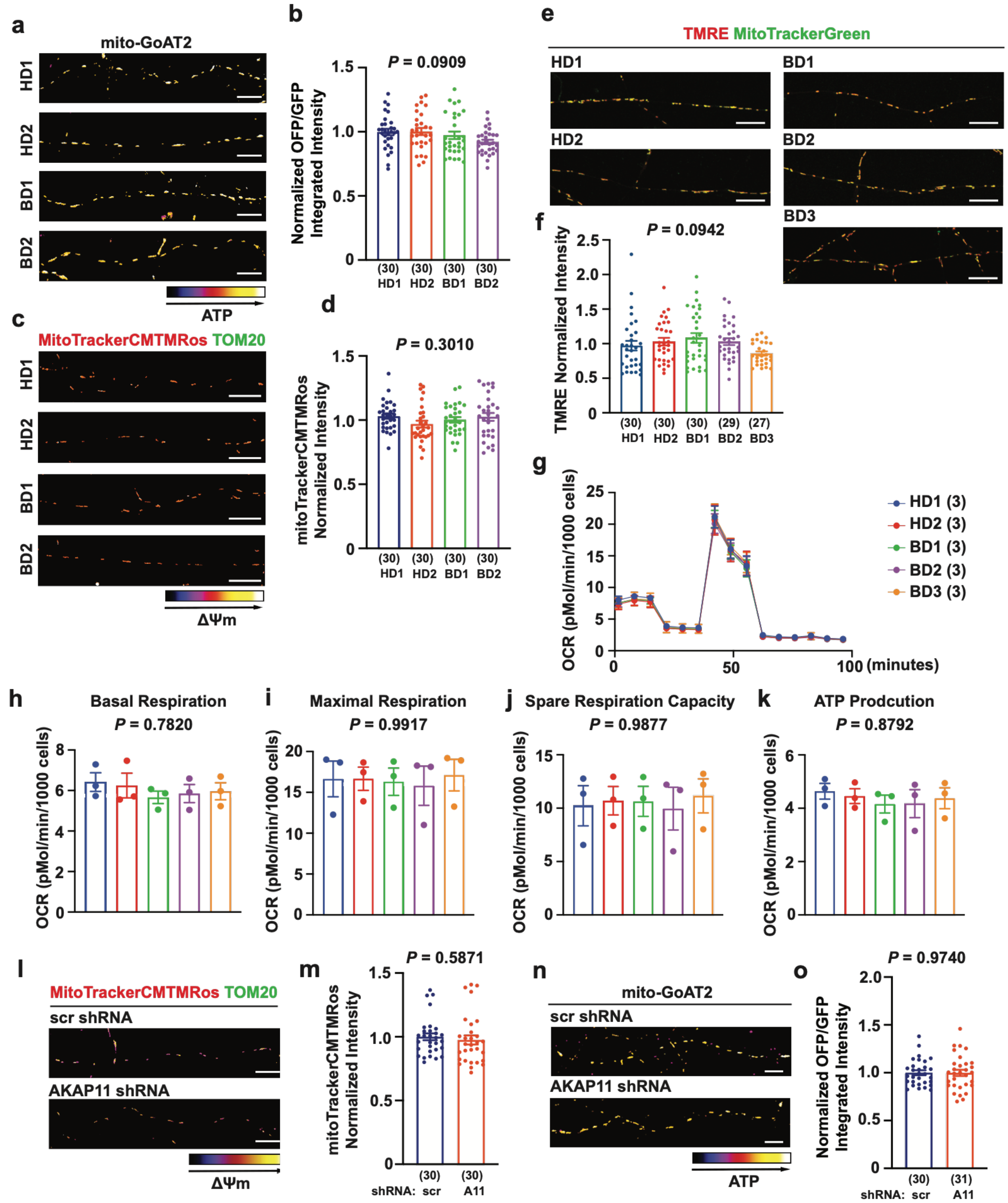
a
mito-GoAT2
HD1
HD2
BD1
BD2
ATP
b
P = 0.0909
Normalized OFP/GFP Integrated Intensity
(30) HD1 (30) HD2 (30) BD1 (30) BD2
c
MitoTrackerCMTMRos TOM20
ΔΨm
d
P = 0.3010
mitoTrackerCMTMRos Normalized Intensity
e
TMRE MitoTrackerGreen
BD3
f
P = 0.0942
TMRE Normalized Intensity
(30) HD1 (30) HD2 (30) BD1 (29) BD2 (27) BD3
g
OCR (pMol/min/1000 cells)
100 (minutes)
HD1 (3)
HD2 (3)
BD1 (3)
BD2 (3)
BD3 (3)
h
Basal Respiration
P = 0.7820
i
Maximal Respiration
P = 0.9917
j
Spare Respiration Capacity
P = 0.9877
k
ATP Prodcution
P = 0.8792
l
scr shRNA
AKAP11 shRNA
m
P = 0.5871
shRNA: scr A11
n
o
P = 0.9740
(30) (31)

Supplementary Fig. 4. Mitochondrial function remains intact in hiPSC-derived neurons from donors with bipolar disorder.

a, b, Mitochondrial ATP levels measured using the mitochondria-targeted ATP sensor Mito-GO-ATeam2. Representative ATP images (the OFP/GFP ratio, **a**) and quantification (**b**) show no significant differences between hiPSC-derived neurons from healthy donors (HD) and donors with bipolar disorder (BD) (HD1 *vs.* BD1: P = 0.8404; HD1 *vs.* BD2: P = 0.1366; HD2 *vs.* BD1: P = 0.8444; HD2 *vs.* BD2: P = 0.1089). Data were obtained from 30 neurons differentiated from each hiPSC line (HD1, HD2, BD1, and BD2) across three independent biological replicates.

c–f, Mitochondrial membrane potential (Δψm) assessed using two independent probes. Representative ratiometric images and quantification using MitoTracker Orange CMTMRos normalized to TOM20 (**c**, **d**) and live imaging with TMRM/MitoTracker Green (**e**, **f**) revealed no significant differences between hiPSC-derived neurons from healthy donors (HD) and donors with bipolar disorder (BD) (for CMTMRos imaging: HD1 *vs.* BD1: P = 0.8491; HD1 *vs.* BD2: P = 0.9987; HD2 *vs.* BD1: P = 0.7967; HD2 *vs.* BD2: P = 0.3992).

g–k, Mitochondrial respiration measured using the Seahorse Mito Stress Test. Oxygen consumption rates (OCRs) were normalized to cell number using Hoechst 33342 staining (**g**). Quantification of basal respiration (**h**), maximal respiration (**i**), spare respiratory capacity (**j**), and ATP production (**k**) showed no significant differences between hiPSC-derived neurons from healthy donors (HD) and donors with bipolar disorder (BD). Data were obtained from three independent biological replicates, each representing an independently performed Seahorse assay using independently prepared neuronal cultures.

l, m, AKAP11-deficient cortical neurons exhibit normal mitochondrial membrane potential. Representative images (**l**) and quantification (**m**) of CMTMRos/TOM20 ratios showed no significant differences between neurons expressing scramble (scr)- and AKAP11-shRNA.

n, o, AKAP11-deficient neurons maintain normal mitochondrial ATP levels. Representative Mito-GO-ATeam2 images (**n**) and quantification (**o**) showed no significant differences between scr- and AKAP11-shRNA groups.

Data are presented as mean ± s.e.m. from the number of neurons indicated in the graphs or parentheses from three independent experiments (**b, d, f, m, o**). Statistical analyses were performed using one-way ANOVA with Tukey's multiple-comparison test (**b**, **d**, **f**, **h-k**) or two-tailed Student's *t*-test (**m**, **o**). Scale bars, 10 μm. Source data are provided as a Source Data file.

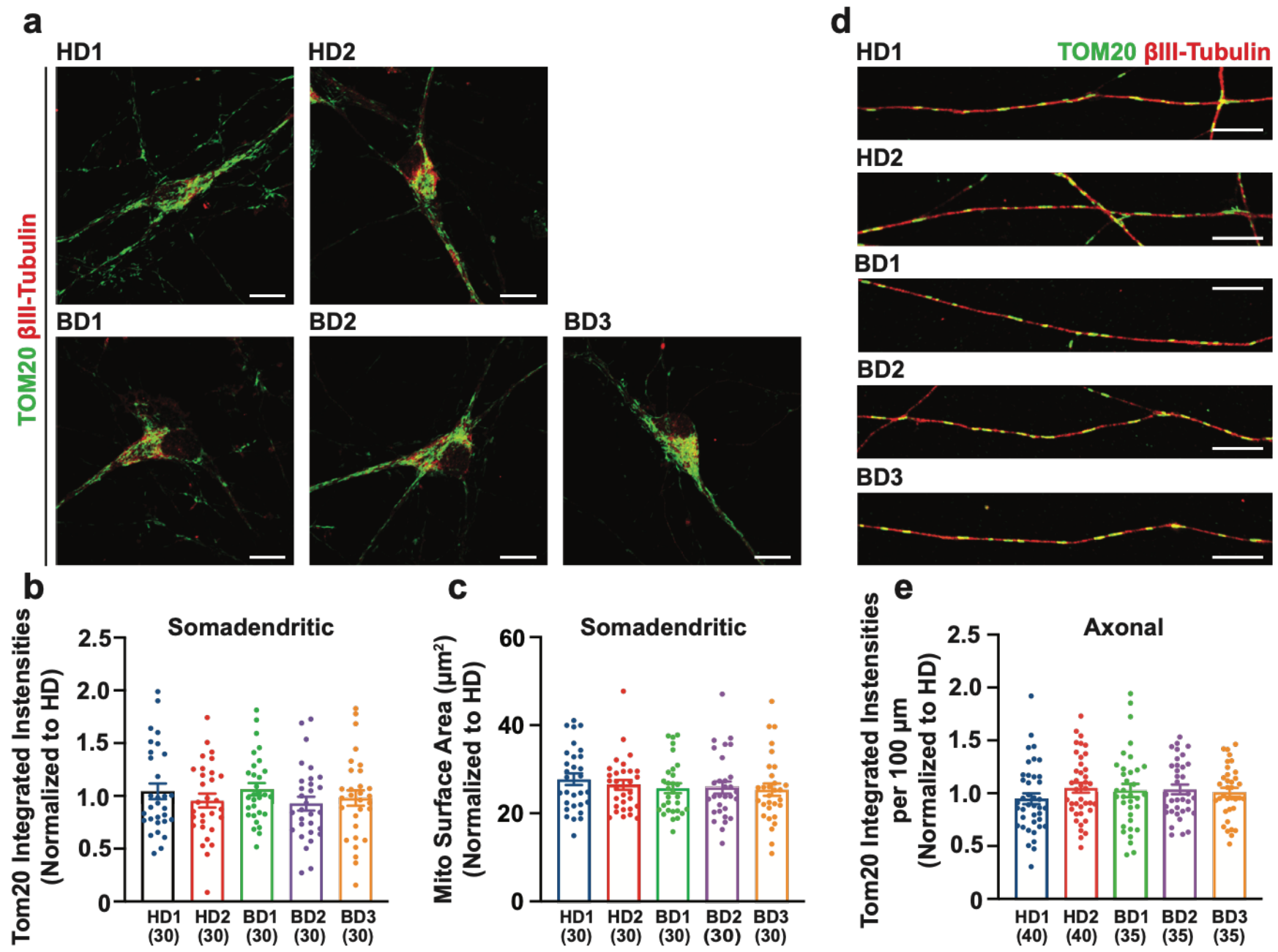

a
HD1
HD2
BD1
BD2
BD3
TOM20 βIII-Tubulin
d
HD1
TOM20 βIII-Tubulin
HD2
BD1
BD2
BD3
b
Somadendritic
Tom20 Integrated Instensities (Normalized to HD)
HD1 (30) HD2 (30) BD1 (30) BD2 (30) BD3 (30)
c
Somadendritic
Mito Surface Area (μm²) (Normalized to HD)
HD1 (30) HD2 (30) BD1 (30) BD2 (30) BD3 (30)
e
Axonal
Tom20 Integrated Instensities per 100 μm (Normalized to HD)
HD1 (40) HD2 (40) BD1 (35) BD2 (35) BD3 (35)

Supplementary Fig. 5. Mitochondrial distribution remains unchanged in hiPSC-derived neurons from donors with bipolar disorder.
Human iPSC-derived cortical neurons at DPI21 were immunostained for TOM20 and β3-tubulin. Mitochondrial distribution was quantified by TOM20 integrated intensity and mitochondrial area in the somatodendritic and axonal compartments. No significant differences were observed between healthy donor (HD) and bipolar disorder (BD) neurons. Data are presented as mean ± s.e.m. from the number of neurons indicated in parentheses from three independent experiments. Statistical analyses were performed using one-way ANOVA with Tukey's multiple-comparison test. Scale bars, 10 μm. Source data are provided as a Source Data file.

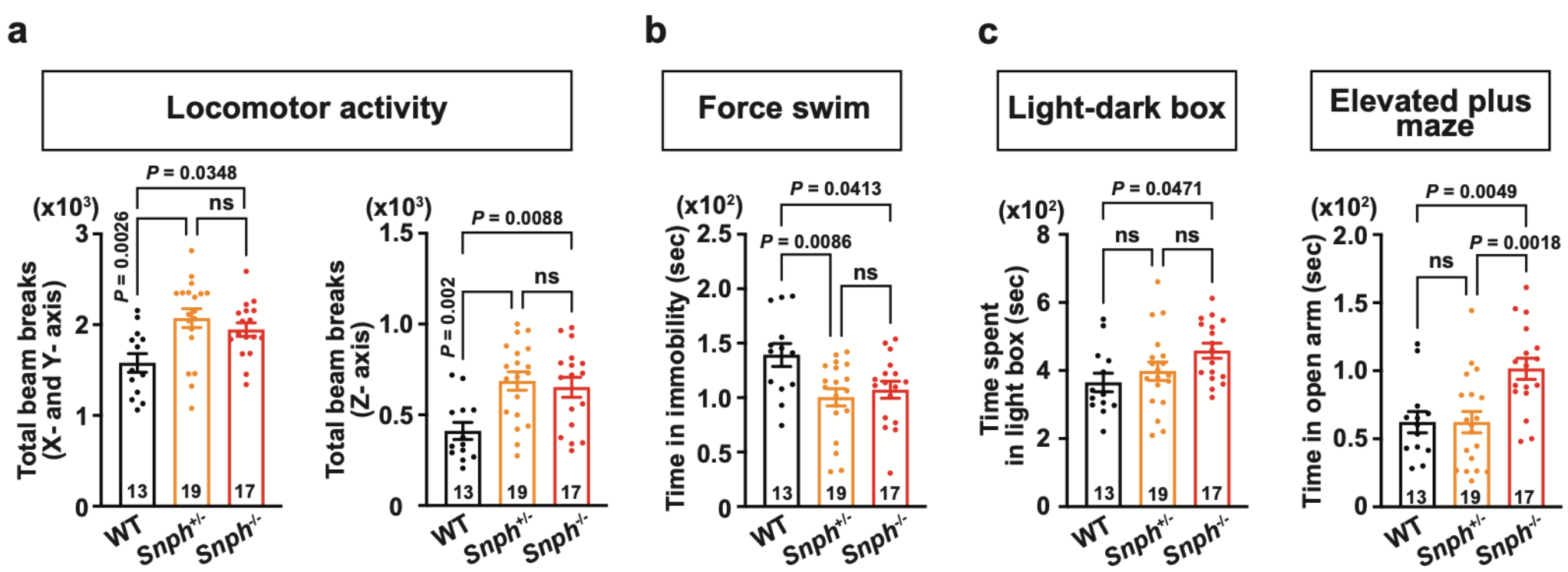

a
Locomotor activity
Total beam breaks (X- and Y- axis)
(x10³)
P = 0.0348
P = 0.0026
ns
Total beam breaks (Z- axis)
P = 0.0088
P = 0.002
ns
b
Force swim
Time in immobility (sec)
(x10²)
P = 0.0413
P = 0.0086
ns
c
Light-dark box
Time spent in light box (sec)
(x10²)
P = 0.0471
ns
ns
Elevated plus maze
Time in open arm (sec)
(x10²)
P = 0.0049
P = 0.0018
ns
13
19
17
WT
Snph+/-
Snph-/-

Supplementary Fig. 6. *Snph*$^{+/-}$ mice partially recapitulate mania-like behavioral traits.

a, Locomotor activity in WT, *Snph*$^{+/-}$, and *Snph*$^{-/-}$ mice (12-14 weeks old) assessed in the open-field test. Horizontal and vertical activity was recorded using a photobeam tracking system along the X- and Y-axis and rearing behavior was recorded by Z-axis beam breaks over 60 min. Both *Snph*$^{+/-}$ and *Snph*$^{-/-}$ mice exhibited increased ambulatory movement (one-way ANOVA, $F_{2, 46}$ = 6.485, P = 0.0033; WT vs. *Snph*$^{+/-}$: P = 0.0026; WT vs. *Snph*$^{-/-}$: P = 0.0348) and rearing behavior compared with WT controls (one-way ANOVA, $F_{2, 46}$ = 7.356, P = 0.0017; WT vs. *Snph*$^{+/-}$: P = 0.0020; WT vs. *Snph*$^{-/-}$: P = 0.0088).

b, Depression-like behavior assessed by the forced swim test. Both *Snph*$^{+/-}$ and *Snph*$^{-/-}$ mice exhibited reduced immobility time relative to WT controls (one-way ANOVA, $F_{2, 46}$ = 5.226, P = 0.0090; WT vs. *Snph*$^{+/-}$: P = 0.0086; WT vs. *Snph*$^{-/-}$: P = 0.0413; *Snph*$^{+/-}$ vs. *Snph*$^{-/-}$: P = 0.8213). indicating decreased depression-like behavior

c, Anxiety-like behavior assessed by the light–dark box and elevated plus maze (EPM). *Snph*$^{-/-}$**,** but not *Snph*$^{+/-}$, mice spent significantly more time in the light compartment (one-way ANOVA, $F_{2, 46}$ = 3.210, P = 0.0495; WT vs. *Snph*$^{+/-}$: P = 0.6514; WT vs. *Snph*$^{-/-}$: P = 0.0471; *Snph*$^{+/-}$ vs. *Snph*$^{-/-}$: P = 0.1995) and in the open arms of the EPM (one-way ANOVA, $F_{2, 46}$ = 8.316, P = 0.0008; WT vs. *Snph*$^{+/-}$: P > 0.9999; WT vs. *Snph*$^{-/-}$: P = 0.0049; *Snph*$^{+/-}$ vs. *Snph*$^{-/-}$: P = 0.0018), indicating reduced anxiety-like behavior.

Data are presented as mean ± s.e.m. from the number of mice indicated in the graphs. Statistical analyses were performed using one-way ANOVA followed by Tukey's multiple-comparison test. Source data are provided as a Source Data file.

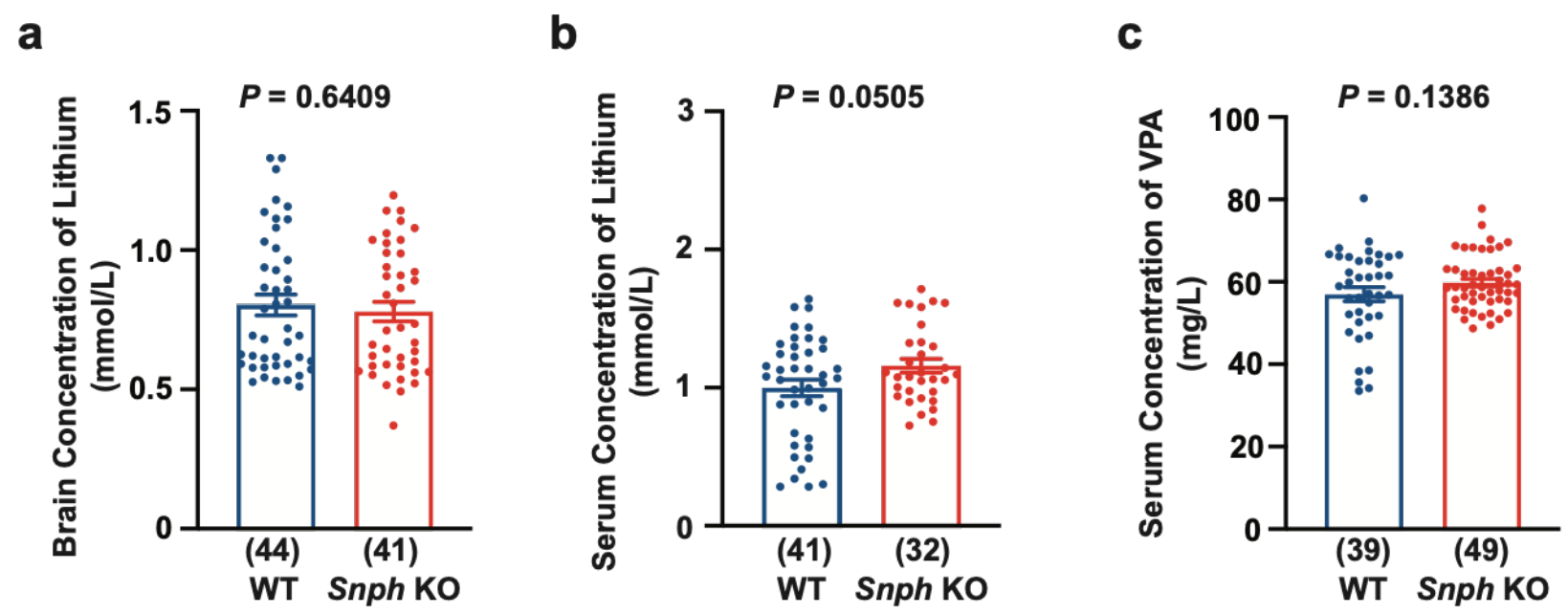
a
P = 0.6409
Brain Concentration of Lithium (mmol/L)
1.5
1.0
0.5
0
(44) WT
(41) Snph KO
b
P = 0.0505
Serum Concentration of Lithium (mmol/L)
3
2
1
0
(41) WT
(32) Snph KO
c
P = 0.1386
Serum Concentration of VPA (mg/L)
100
80
60
40
20
0
(39) WT
(49) Snph KO

Supplementary Fig. 7: Lithium and valproic acid (VPA) concentrations in treated mouse brains and serum.
Lithium concentrations were measured by inductively coupled plasma mass spectrometry (ICP-MS) in brain homogenates (**a**) and serum (**b**) from WT and *Snph*$^{-/-}$ mice after 4 weeks of intraperitoneal lithium treatment. Serum VPA concentrations (**c**) were measured by low-resolution electrospray ionization mass spectra (LRESIMS) after 4 weeks of intraperitoneal VPA treatment. No significant differences in lithium or VPA concentrations were detected between WT and *Snph*$^{-/-}$ mice. Data are presented as mean ± s.e.m. from the number of animals indicated in the graphs. Statistical analyses were performed using two-tailed Student's *t*-test. *n.s.*, not significant. Source data are provided as a Source Data file.

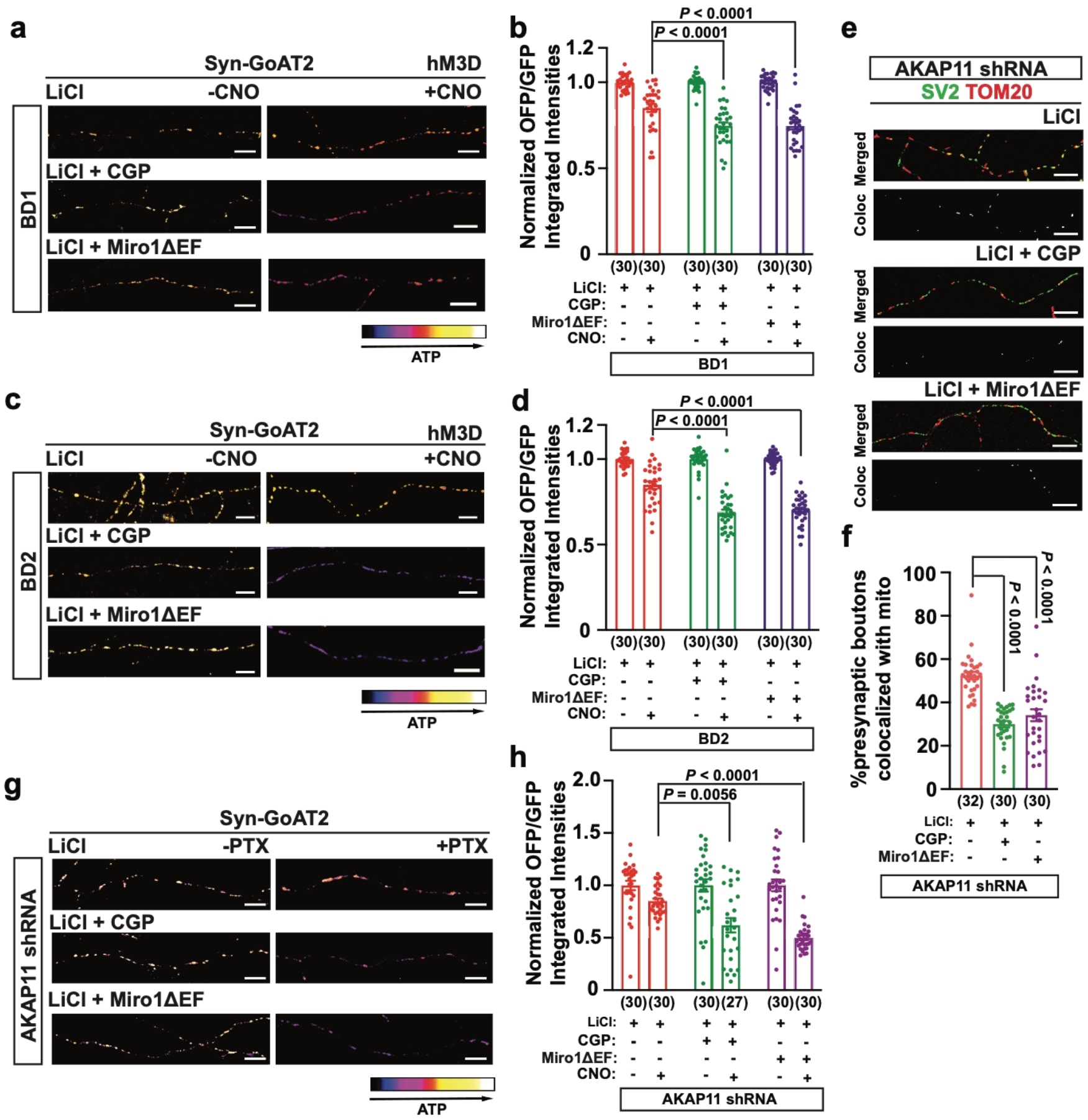
a
Syn-GoAT2
hM3D
LiCl
-CNO
+CNO
BD1
LiCl + CGP
LiCl + Miro1ΔEF
ATP
b
P < 0.0001
P < 0.0001
Normalized OFP/GFP Integrated Intensities
(30)(30) (30)(30) (30)(30)
LiCl:
CGP:
Miro1ΔEF:
CNO:
BD1
c
Syn-GoAT2
hM3D
LiCl
-CNO
+CNO
BD2
LiCl + CGP
LiCl + Miro1ΔEF
ATP
d
P < 0.0001
P < 0.0001
Normalized OFP/GFP Integrated Intensities
(30)(30) (30)(30) (30)(30)
LiCl:
CGP:
Miro1ΔEF:
CNO:
BD2
e
AKAP11 shRNA
SV2 TOM20
LiCl
Merged
Coloc
LiCl + CGP
LiCl + Miro1ΔEF
f
%presynaptic boutons colocalized with mito
P < 0.0001
P < 0.0001
(32) (30) (30)
LiCl:
CGP:
Miro1ΔEF:
AKAP11 shRNA
g
Syn-GoAT2
LiCl
-PTX
+PTX
AKAP11 shRNA
LiCl + CGP
LiCl + Miro1ΔEF
ATP
h
P < 0.0001
P = 0.0056
Normalized OFP/GFP Integrated Intensities
(30)(30) (30)(27) (30)(30)
LiCl:
CGP:
Miro1ΔEF:
CNO:
AKAP11 shRNA

Supplementary Fig. 8. Lithium restores synaptoenergetics in bipolar disorder-associated neurons through NCLX- and Miro-mediated Ca^{2+} signaling.
a-d, LiCl-mediated restoration of presynaptic ATP levels in hiPSC-derived cortical neurons from donors with bipolar disorder (BD) requires NCLX activity and Miro Ca^{2+} binding. BD1 (**a, b**) and BD2 (**c, d**) neurons expressing hM3D(Gq) were transfected with Syn-GoATeam2 alone or together with myc-Miro1ΔEF at DPI17, treated with DMSO or LiCl (100 μM) for 4 days, and incubated with DMSO or the NCLX inhibitor CGP37157 (2 μM) for 4 hours before CNO stimulation (10 μM) at DPI21. Representative ATP ratio images and quantification show that inhibition of NCLX or expression of Miro1ΔEF abolishes the LiCl-mediated increase in presynaptic ATP.
e, f, LiCl-mediated restoration of presynaptic mitochondrial retention in AKAP11-deficient mouse cortical neurons requires NCLX activity and Miro Ca^{2+} binding. Neurons expressing AKAP11-shRNA were transfected with myc-Miro1ΔEF at DIV9 and treated with DMSO or LiCl (100 μM) for 4 days (DIV10-14). Neurons were then incubated for 4 hours with DMSO or CGP37157 (2 μM) prior to co-immunostaining for SV2 and TOM20 at DIV14. Representative images (**e**) and quantification (**f**) of SV2/TOM20 colocalization show that the effects of LiCl are blocked by CGP37157 or Miro1ΔEF expression.
g, h, LiCl-mediated restoration of presynaptic ATP levels in AKAP11-deficient neurons requires NCLX activity and Miro Ca^{2+} binding. Representative ATP ratio images (**g**) and quantification (**h**) show that LiCl-induced ATP recovery is prevented by CGP37157 or Miro1ΔEF expression.
Data are presented as mean ± s.e.m. from the number of neurons indicated in parentheses from three independent experiments. Statistical analyses were performed using one-way ANOVA with Dunnett's multiple-comparison test. Scale bars, 10 μm. Source data are provided as a Source Data file.

	Sex	Age	Description
HD1	Male	23	Unaffected
HD2	Female	20	Unaffected
BD1	Male	20	Type I Bipolar
BD2	Female	23	Type I Bipolar
BD3	Female	28	Type I Bipolar

Supplementary Table 1. Human donor information.
Human fibroblasts from two healthy donors (HD1, HD2) and three donors with bipolar disorder (BD1, BD2, BD2) were obtained from the Coriell Institute for Medical Research and reprogrammed into induced pluripotent stem cells (iPSCs), which were subsequently differentiated into cortical neurons.
