# Supplementary material for: Enhanced axonal mitochondrial motility and neural activity-induced energy deficits destabilize synaptic transmission in models of bipolar disorder
Source: Nat Commun. 2026 Aug 22;17:10159. doi: 10.1038/s41467-026-76722-x (PMC13601522; doi:10.1038/s41467-026-76722-x)
Supplement: Supplementary file 2 [file 41467_2026_76722_MOESM2_ESM.pdf]

nature portfolio

Corresponding author(s): Zu-Hang Sheng

Last updated by author(s): 08/04/2026

Reporting Summary

Nature Portfolio wishes to improve the reproducibility of the work that we publish. This form provides structure for consistency and transparency in reporting. For further information on Nature Portfolio policies, see our Editorial Policies and the Editorial Policy Checklist.

Statistics

For all statistical analyses, confirm that the following items are present in the figure legend, table legend, main text, or Methods section.

n/a	Confirmed	
☐	☒	The exact sample size (*n*) for each experimental group/condition, given as a discrete number and unit of measurement
☐	☒	A statement on whether measurements were taken from distinct samples or whether the same sample was measured repeatedly
☐	☒	The statistical test(s) used AND whether they are one- or two-sided<br>*Only common tests should be described solely by name; describe more complex techniques in the Methods section.*
☐	☒	A description of all covariates tested
☐	☒	A description of any assumptions or corrections, such as tests of normality and adjustment for multiple comparisons
☐	☒	A full description of the statistical parameters including central tendency (e.g. means) or other basic estimates (e.g. regression coefficient) AND variation (e.g. standard deviation) or associated estimates of uncertainty (e.g. confidence intervals)
☐	☒	For null hypothesis testing, the test statistic (e.g. *F*, *t*, *r*) with confidence intervals, effect sizes, degrees of freedom and *P* value noted<br>*Give P values as exact values whenever suitable.*
☒	☐	For Bayesian analysis, information on the choice of priors and Markov chain Monte Carlo settings
☒	☐	For hierarchical and complex designs, identification of the appropriate level for tests and full reporting of outcomes
☒	☐	Estimates of effect sizes (e.g. Cohen's *d*, Pearson's *r*), indicating how they were calculated

Our web collection on statistics for biologists contains articles on many of the points above.

Software and code

Policy information about availability of computer code

Data collection	ZEN (Zeiss), pCLAMP (Molecular Devices), ANY-maze (ANY-maze), Topscan behavioral tracking software (NIMH Rodent Behavioral Core Facility), Wave Controller (Agilent)
Data analysis	ImageJ2, Prism 9 (GraphPad), Seahorse Analytics (Agilent), Clampfit10.2

For manuscripts utilizing custom algorithms or software that are central to the research but not yet described in published literature, software must be made available to editors and reviewers. We strongly encourage code deposition in a community repository (e.g. GitHub). See the Nature Portfolio guidelines for submitting code & software for further information.

Data

Policy information about availability of data

All manuscripts must include a data availability statement. This statement should provide the following information, where applicable:

- Accession codes, unique identifiers, or web links for publicly available datasets
- A description of any restrictions on data availability
- For clinical datasets or third party data, please ensure that the statement adheres to our policy

All data supporting the findings of this study are provided in the Supplementary Information/Source Data file. Because of their very large file size, the original microscopy images and time-lapse live images, and videos recording behavioral phenotypes cannot be publicly deposited and are available from the corresponding author upon request. Any additional information required to reanalyze the data reported in this paper is available from the corresponding author upon request.

Research involving human participants, their data, or biological material

Policy information about studies with human participants or human data. See also policy information about sex, gender (identity/presentation), and sexual orientation and race, ethnicity and racism.

Reporting on sex and gender	N/A
Reporting on race, ethnicity, or other socially relevant groupings	N/A
Population characteristics	N/A
Recruitment	N/A
Ethics oversight	N/A

Note that full information on the approval of the study protocol must also be provided in the manuscript.

Field-specific reporting

Please select the one below that is the best fit for your research. If you are not sure, read the appropriate sections before making your selection.

☒ Life sciences ☐ Behavioural & social sciences ☐ Ecological, evolutionary & environmental sciences

For a reference copy of the document with all sections, see nature.com/documents/nr-reporting-summary-flat.pdf

Life sciences study design

All studies must disclose on these points even when the disclosure is negative.

Sample size	For cell-based experiments, at least three independent biological replicates were performed, with sample sizes based on the variability and effect sizes observed in our previous studies using similar assays (Li et al., Nature Metabolism, 2020). For mouse behavioral experiments, sample sizes were based on our previous studies using similar methodologies (Xiong et al., Molecular Psychiatry, 2021). No formal statistical method was used to predetermine sample size. The exact sample size for each experiment is provided in the corresponding figure legends.
Data exclusions	Neurons with obvious defective morphology were excluded from the analysis.
Replication	All replicates are biological replicates. The number of replicates is indicated in the legend of each Figure.
Randomization	Sample allocation was random.
Blinding	Group allocation was not blinded.

Reporting for specific materials, systems and methods

We require information from authors about some types of materials, experimental systems and methods used in many studies. Here, indicate whether each material, system or method listed is relevant to your study. If you are not sure if a list item applies to your research, read the appropriate section before selecting a response.

Materials & experimental systems

n/a	Involved in the study	
☐	☒	Antibodies
☐	☒	Eukaryotic cell lines
☒	☐	Palaeontology and archaeology
☐	☒	Animals and other organisms
☒	☐	Clinical data
☒	☐	Dual use research of concern
☒	☐	Plants

Methods

n/a	Involved in the study	
☒	☐	ChIP-seq
☒	☐	Flow cytometry
☒	☐	MRI-based neuroimaging

Antibodies

Antibodies used	anti-SV2 (DSHB, cat#SV2); anti-betaIII-Tubulin (BioLegend, cat#802001); anti-PSD95 (ThermoFisher, cat#MA1-045); anti-TOM20

Antibodies used	(Santa Cruz, cat#sc-11415); anti-GAPDH (Millipore, cat#CB1001); anti-AKAP11 (Novus, cat#H00011215-A01); anti-SNPH (Abcam, cat#ab192605); anti-synaptophysin (Santa Cruz, cat#sc-9116; anti-c-Fos (Santa Cruz, cat#sc-271243); anti-HA-Tag (Cell Signaling; cat#3724); TRA-1-60 Alexa Fluor 488 (Thermo Fisher, cat#A25618); Goat anti-Mouse (H+L) Cross-Adsorbed Secondary Antibody, Alexa Fluor 488 (Thermo Fisher; cat#A-11001); Goat anti-Mouse (H+L) Cross-Adsorbed Secondary A;ntibody, Alexa Fluor 546 (Thermo Fisher; cat#A-11008); Goat anti-Rabbit (H+L) Cross-Adsorbed Secondary Antibody, Alexa Fluor 488 (Thermo Fisher; cat#A-11001); Goat anti-Mouse (H+L) Cross-Adsorbed Secondary Antibody, Alexa Fluor 488 (Thermo Fisher; cat#A-11003); Goat anti-Rabbit (H+L) Cross-Adsorbed Secondary Antibody, Alexa Fluor 488 (Thermo Fisher; cat#A-11010); Mouse IgG HRP Linked Whole Antibody (GE Healthcare, cat#N931); Rabbit IgG HRP Linked Whole Antibody (GE Healthcare, cat#N934).
Validation	All primary antibodies were validated by the manufacturers.

Eukaryotic cell lines

Policy information about cell lines and Sex and Gender in Research

Cell line source(s)	Human fibroblast GM05915, GM05992, GM05224, GM05215, GM07219 (Coriell Institute)
Authentication	No authentication information is provided by the vendor.
Mycoplasma contamination	Cell lines were not tested for mycoplasma after acquisition.
Commonly misidentified lines (See ICLAC register)	No commonly misidentified cell lines were used in this study.

Animals and other research organisms

Policy information about studies involving animals; ARRIVE guidelines recommended for reporting animal research, and Sex and Gender in Research

Laboratory animals	Male WT, Snph+/-, and Snph-/- transgenic mice maintained on C57BL/6J background at 12-14 week-old were randomly assigned to behavioral analyses. A total 151 WT, 19 Snph+/-, 170 Snph-/- trangenic mice were used in the study. The exact number of animal used for each experiment is indicated in the legend of each figure.
Wild animals	No wild animals were used in the study.
Reporting on sex	Behavioral experiments were conducted in male mice to maintain a homogeneous experimental cohort during the initial behavioral characterization. Future studies including both sexes will be required to determine the generalizability of these findings.
Field-collected samples	The study did not include field-collected samples
Ethics oversight	All animal experiments were performed in accordance with the National Institutes of Health Guide for the Care and Use of Laboratory Animal. Animal study protocol (#1303) was approved by the Animal Care and Use Committee of the National Institute of Neurological Disorders and Stroke (NINDS) and the National Institute on Deafness and other Communication Disorders (NIDCD) and National Center for Complementary and Integrative Health (NCCIH).

Note that full information on the approval of the study protocol must also be provided in the manuscript.

Plants

Seed stocks	N/A
Novel plant genotypes	N/A
Authentication	N/A
